# Matrix factorization and transfer learning uncover regulatory biology across multiple single-cell ATAC-seq data sets

**DOI:** 10.1093/nar/gkaa349

**Published:** 2020-05-11

**Authors:** Rossin Erbe, Michael D Kessler, Alexander V Favorov, Hariharan Easwaran, Daria A Gaykalova, Elana J Fertig

**Affiliations:** Johns Hopkins University, Baltimore, MD, USA; Johns Hopkins University, Baltimore, MD, USA; Johns Hopkins University, Baltimore, MD, USA; Vavilov Institute of General Genetics, Moscow, Russia; Johns Hopkins University, Baltimore, MD, USA; Johns Hopkins University, Baltimore, MD, USA; Johns Hopkins University, Baltimore, MD, USA

## Abstract

While the methods available for single-cell ATAC-seq analysis are well optimized for clustering cell types, the question of how to integrate multiple scATAC-seq data sets and/or sequencing modalities is still open. We present an analysis framework that enables such integration across scATAC-seq data sets by applying the CoGAPS Matrix Factorization algorithm and the projectR transfer learning program to identify common regulatory patterns across scATAC-seq data sets. We additionally integrate our analysis with scRNA-seq data to identify orthogonal evidence for transcriptional regulators predicted by scATAC-seq analysis. Using publicly available scATAC-seq data, we find patterns that accurately characterize cell types both within and across data sets. Furthermore, we demonstrate that these patterns are both consistent with current biological understanding and reflective of novel regulatory biology.

## INTRODUCTION

The Assay for Transposase Accessible Chromatin (ATAC-seq) subjects DNA to a hyperactive transposase in order to tag euchromatic regions of the genome for sequencing. ATAC-seq thus provides a quantitative estimate of genome-wide chromatin accessibility, and can be used to infer which genomic regions are most likely to interact directly with proteins and other biologically relevant molecules (1,2). Specifically, accessibility at enhancers and promoters has considerable influence on the binding of transcription factors (TFs) and other transcriptional machinery (3). Quantification of accessibility at these regions enables the characterization of the regulatory biology that defines cell types and samples of interest (1,2).

ATAC-seq data is often summarized by binning reads into data-defined genomic regions of frequent accessibility (generally termed peaks) or by aggregating the reads that contain annotated DNA motifs (e.g. transcription factor binding sites), which are collectively the targets of defined trans-acting factors (e.g. transcription factors) (4). Aggregating reads in these ways allows for a comparison of accessibility variation between samples and inference of the chromatin landscape of cell populations. However, the functional annotations available for these features are often incomplete (as explored in detail by (5)), which can present significant challenges in the interpretation of ATAC-seq data and limit the integration of accessibility information across data sets. Furthermore, the high dimensionality and extreme sparsity of single cell ATAC-seq data (scATAC-seq) significantly compounds these analytic challenges, and further limits interpretation (6).

Therefore, computational methods are necessary to determine the patterns of accessibility that differentiate the regulatory biology associated with disparate cell populations in scATAC-seq data. Current tools for scATAC-seq analysis robustly cluster and annotate cell types. For example, ChromVAR, BROCKMAN, *Cusanovitch2018* and scABC (7–10) all output both clustering and inferred transcription factor binding within clusters, using clustering accuracy as their primary metric to evaluate efficacy. SnapATAC and cisTopic additionally provide the ability to query upregulated pathways from scATAC-seq data, but are still most strongly oriented towards the goal of effectively differentiating cell populations (6,11). These methods provide effective tools for the analysis of individual scATAC-seq data, but require further extension to integrate the information learned from multiple scATAC-seq experiments or multiple sequencing modalities and thus use the multiple lines of evidence available to inform conclusions.

We develop a framework to enable cross-study and cross-platform analysis of multiple scATAC-seq data sets through the application of the Bayesian Non-Negative Matrix Factorization algorithm, CoGAPS, (12,13) in conjunction with the transfer learning program projectR (14,15). We demonstrate that CoGAPS simultaneously identifies robust cell types, upregulated pathways, and TF activity from scATAC-seq data. Notably, the projectR transfer learning method allows for recognition of the learned signatures of regulatory biology that we identify with CoGAPS in other datasets. Finally, we use matched RNA-seq data to provide orthogonal evidence for candidate regulatory mechanisms identified by our scATAC-seq analysis method. This workflow facilitates the development of consensus accessibility signatures for cellular populations using multiple data sets and data modalities. Furthermore, we demonstrate that combined CoGAPS analysis of scATAC-seq and scRNA-seq identifies novel biology, such as the association of the transcription factor Hnf4a with mammalian cardiac development.

## MATERIALS AND METHODS

### ATAC-CoGAPS pipeline

The ATAC-CoGAPS software is freely available as an R package from https://github.com/FertigLab/ATACCoGAPS with current release archived on Zenodo at https://zenodo.org/record/3701789. This software package includes functions for preprocessing of scATAC-seq data to run the CoGAPS algorithm (version ≥ 3.5.13), as well as functions for subsequent analysis of the results. Each of the steps taken to perform the standard ATAC-CoGAPS workflow are available from https://rossinerbe.github.io/ATACCoGAPS_Tutorial with the current release archived on Zenodo at https://zenodo.org/badge/latestdoi/216057447. All analysis code and filtered input data used to produce the results described in this work are available from https://github.com/rossinerbe/ATACCoGAPS-Analysis-Code with the current release archived on Zenodo at https://zenodo.org/badge/latestdoi/215837627.

**Figure 1.**
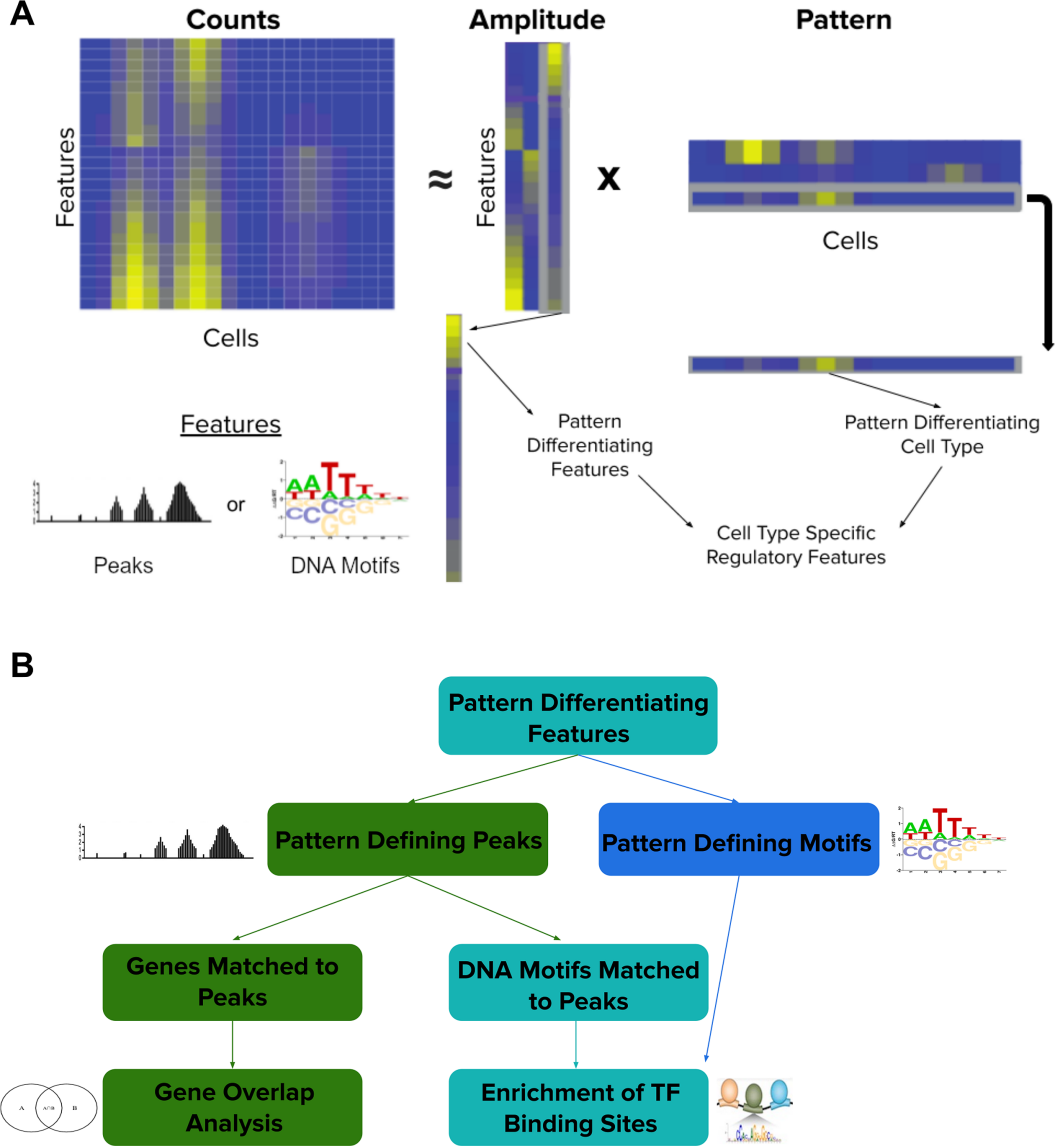
(**A**) Diagram of Non-negative Matrix Factorization as applied to scATAC-seq data by ATAC-CoGAPS. The Counts matrix (features by cells) is factorized into the Amplitude matrix (features by learned patterns) and the Pattern matrix (learned patterns by cells). The patterns in the Pattern matrix differentiate cell populations, while the same patterns in the Amplitude matrix reveal the differentially accessible features of those cell types. These cell type specific patterns of accessibility can then be used to learn regulatory features that differ across cell populations. (**B**) Diagram of the analysis approach applied for cell type associated features found by CoGAPS. Features used to produce the input count matrix can be either accessible peaks or DNA motifs. Pattern defining peaks identified by CoGAPS are either matched to genes for gene overlap analysis or matched to DNA motifs to infer TF binding potential. Pattern defining motifs are matched to enriched to TFs, likewise to infer accessible binding sites and thus TF activity in identified cell populations.

Input reads from a scATAC-seq experiment are summarized into some feature space (peaks, DNA motifs, etc.) and into an input count matrix, features by cells. Specific preprocessing steps are outlined in the analysis code linked above. Next, the count matrix is input to the R/Bioconductor package CoGAPS (version 3.5.8 for all analyses conducted in this work). CoGAPS employs a sparse, Bayesian non-negative matrix factorization algorithm to decompose the scATAC-seq count matrix **C**, features by cells, into an Amplitude matrix **A**, features by learned patterns, and a Pattern matrix **P**, learned patterns by cells as described in (12) and (13) (and visualized in Figure 1A). This factorization is performed according to the model:

$$\begin{equation*}{\rm{p}}\left( {{\rm{A,P|C,\Sigma }}} \right) \propto {\rm{p}}\left( {{\rm{C|A,P,\Sigma }}} \right){\rm{p}}\left( {\rm{A}} \right){\rm{p}}\left( {\rm{P}} \right)\end{equation*}$$

where **p(C|A,P,Σ)** is a univariate normal distribution for each element of **C** with mean given by the matrix product **AP** and **Σ** represents the standard deviation of each element in **C**. CoGAPS uses an atomic prior (16) for both **p(A)** and **p(P)** to model the sparsity and non-negativity of the input count matrix. Briefly, the atomic prior element of the **A** and **P** matrices is either set to zero or made to follow a gamma distribution based on sampling from a Poisson prior. The Poisson prior is used as the shape parameter of the gamma distribution and a fixed hyperparameter for all matrix elements is used as the scale parameter of the gamma distribution. The use of these distributions allows for Gibbs sampling. For a more detailed description of CoGAPS and derivation of the sampling algorithm, see (12,13,17). The primary parameters for the application of CoGAPS are the feature level summarizations used to obtain the count matrix **C** and number of learned patterns, described in further detail below. To account for sparsity, we filter the input count matrix **C** to remove any feature or cell that is more than 99% zero.

The next steps of the ATAC-CoGAPS analysis framework then focuses on the output **A** and **P** matrices. Unless otherwise noted, all steps are functionalized within the ATACCoGAPS package and all outside packages used are wrapped within ATACCoGAPS functions (see the workflow at https://rossinerbe.github.io/ATACCoGAPS_Tutorial for detailed implementation with code). We first evaluate the results object from CoGAPS by plotting the Pattern matrix **P** (learned patterns by cells) to determine which patterns differentiate which cell populations. Annotations of patterns to cell populations are made using the PatternMarker statistic (described by (13)) to determine the pattern each cell is most defined by, thereby clustering cells to each pattern. Whereas many functional annotations of factorizations rely on the absolute weights per cell in the **P** matrix, the PatternMarker statistic computes the extent to which that weight is uniquely high in one specific pattern thereby enabling this annotation. Alternatively, if *a priori* determined cell populations are known (e.g. by fluorescence activated cell sorting) we can determine which of these populations have significant signal in a pattern by calling the pairwise.wilcox.test R function for each pattern (not functionalized in ATAC-CoGAPS) instead of the reliance on the data-driven markers from this PatternMarker statistic. The Adjusted Rand Index is used to quantify the overall clustering of CoGAPS on the Schep *et al.* data set (7) using the pattern to cell line annotations listed in Supplemental Table S2. Once these correspondences of pattern to cell type are annotated, we can then turn to the Amplitude matrix **A** (features by learned patterns). We apply the PatternMarker statistic to find the accessible features that most strongly contribute to each pattern, and thus most define the cell population they distinguish. The number of features used in these analyses is determined by thresholding of the PatternMarker statistic such that the feature is assigned to the pattern for which its association is scored most highly (13). The PatternMarker peaks are further ranked for each pattern, and options are included to only use the most highly ranked peaks for analysis. All peaks are used by default and in all analyses presented in this work.

Analysis of the amplitude matrix **A** also depends critically on functional annotation. If peaks are used as summarization, we first match peaks to genes or gene promoters within those regions using the GenomicRanges R package version 1.36.1 (18). We then find enrichment of those genes within known pathways from MSigDB (in this work we demonstrate this capability using Hallmark Pathways v7.0) (19,20) using the GeneOverlap R package version 1.20.0 (21). *P*-values from the enrichment test are FDR corrected using the Benjamini–Hochberg procedure.

Additionally, peaks are matched to DNA motifs with potential TF binding sites using the motifmatchR Package version 1.6.0 (7). TFs with common possible binding sites in multiple PatternMarker accessible regions are returned, along with functional annotations, so the biological plausibility of a TF’s activity in a particular cell population based on known function can be considered alongside the enrichment results. Next, the accessibility of the peaks overlapping with the TF gene itself is evaluated relative to the general accessibility of peaks for that cell population to provide evidence as to whether the TF itself is expressed. For each peak that overlaps with the TF gene, the number of cells with accessible reads are counted within the cell population of interest. This number is averaged for all peaks overlapping the TF gene and then this average is divided by the average quantity of accessible cells for all peaks in the cell population. The resultant fold accessibility value is not intended as a precise quantification, but rather an approximate guide to assess whether a TF gene is generally accessible in a particular cell population.

If the data is summarized to motifs before running CoGAPS using ATACCoGAPS preprocessing functions (which employ motifmatchR for motif matching), the downstream analysis is performed similar to the above. Common TF bindings are returned and assessed for relative accessibility to determine whether the TFs are likely to be themselves expressed in the cell population. Relative accessibility of the TF genes is calculated as described previously.

Learned patterns can be projected into other data sets to determine if the signatures identifying cell populations within one data set apply more generally. We use the projectR package version 1.0.0 (14,15) to perform this analysis. If we use a peak feature space for transfer learning, peaks in the target data set must be matched to peaks in the source data set to project the patterns learned in the source data set. We use the set of all peaks that have any overlap between the two sets as the features we project from and into. If we instead apply DNA motifs as the feature space, all motifs that occur in both data sets are used for projection.

We apply CoGAPS to scRNA-seq data in order to validate candidate TFs identified by scATAC-seq analysis. First, patterns that distinguish the same cell populations are identified. Then, the PatternMarker statistic is used to rank the scRNA-seq genes most associated to each pattern. The TFs identified as described above in scATAC-seq are matched to annotations from the TRRUST database version 2 (22) which list the genes the TFs are known to regulate. These gene sets are compared to the scRNA-seq CoGAPS based gene rankings by gene set enrichment analysis implemented with the fgsea R package version 1.10.1 (23). TFs with statistically significant enrichment (FDR corrected using the Benjamini-Hochberg Procedure) of the genes they are known to regulate are considered to be supported by multimodal analysis.

### CoGAPS Hyperparameters

All CoGAPS analyses presented in this manuscript are performed with CoGAPS version 3.70.0. Factorizations are performed in parallel across random subsets of features using the genome-wide option (13) (which should be used unless there are more cells than the features, in which case the single-cell option should be used instead) and 10,000 iterations. The only remaining free input parameter for CoGAPS is then the number of patterns, **n**, to learn from the data. The input matrix is features by cells, the Amplitude matrix is features by **n**, and the Pattern matrix is **n** by cells. We note that selecting the number of patterns for unsupervised learning methods is an open question in machine learning. Previously, the goodness of fit of the model from the factorization relative to the data across a range of values of **n** has been shown to provide a performance metric for selection of **n** (24,25). *A priori* knowledge of the set of conditions or populations each cell derives from can provide an initial heuristic for the selection of **n**. Several CoGAPS runs can be performed in parallel to test different numbers of patterns. After these CoGAPS runs, a Chi-squared statistic can be computed on the output of the **A** and **P** matrices relative to **C** to determine the goodness of fit of the results and provide numerical guidance on the question of how well different numbers of patterns fit the data.

### Comparison to other analysis methods

We perform additional comparisons of ATACCoGAPS to gradient-based NMF with the scikit-learn Python package was used to run Non-negative Double Singular Value Decomposition Matrix Factorization from (5), cisTopic version 0.2.2 (6), and Seurat version 3.1.4 (26). TF enrichment analysis and functional annotations were performed as recommended for each method, specifically using https://satijalab.org/signac/articles/motif_vignette.html for Seurat and https://github.com/aertslab/cisTopic for cisTopic. To produce a direct comparison of the pathways each algorithm associates with each cell type, here we employ the same package to identify GO terms from peak regions in CoGAPS and cisTopic (rGREAT version 1.16.1 (27)), and use the structure of cisTopic's result plotting function to visualize the results for both algorithms. We additionally test the results of rGREAT against the genomic region annotation-based method we use in this work. To find enrichment for MSigDb Hallmark Pathways with GREAT, we run the algorithm to find the genes it significantly associates with the PatternMarker peaks for each pattern and use those sets of genes for Gene Overlap analysis with the Hallmark Pathways.

All code for the methods comparisons performed in this work is available at https://github.com/rossinerbe/ATACCoGAPS-Analysis-Code.

### Public data

This study presents analyses on publicly available scATAC-seq data from (7) (GSE99172), (28) (GSE96769) and (29) (https://github.com/loosolab/cardiac-progenitors on 8 July 2019). In all cases, data were obtained at peak summary (see papers for alignment and peak calling details). Both the Schep *et al.* 2017 and the Buenrostro *et al.* 2018 scATAC-seq datasets were downloaded with peaks of equal width. The peaks called for the Jia *et al.* 2018 data set were not of equal width, so counts were normalized by dividing the values of each peak by its nucleotide width. Motif counts were obtained using ATACCoGAPS software to convert peak counts to motif counts. The scRNA-seq data set from Jia *et al.* 2018 contains matched single cells to the scATAC-seq dataset. These data were also obtained from https://github.com/loosolab/cardiac-progenitors on 8 July 2019 as normalized counts. Prior to running CoGAPS, all peaks and cells that were more than 99% sparse were filtered out of the data (32 789 peaks and 528 cells for the Schep et al. data set and none for the Jia *et al.* data set (as it was pre-filtered by Jia *et al.*)). Input dimensions for the Schep et al. data set are 90 300 peaks by 1392 cells. The Buenrostro et al. data set was input to ProjectR with filtered dimensions of 62 387 peaks by 1331 cells. The Jia *et al.* was input at 67 368 peaks by 695 cells for the scATAC-seq data and 12 048 genes by 236 cells for the scRNA-seq data. The filtered data sets used as input to CoGAPS are all available at https://github.com/rossinerbe/ATACCoGAPS-Analysis-Code/tree/master/data.

CoGAPS was run for 7, 13 and 18 patterns in this work on the Schep *et al.* (2017) data set. CoGAPS was run for seven patterns on the scATAC-seq data set and six patterns on the scRNA-seq data from Jia *et al.* (2018).

### Computational runtime

CoGAPS runs were performed on Amazon AWS Batch servers. The Schep *et al.* (2017) data set was run across nine cores and ran in 2–8 h for different numbers of patterns selected. The Jia *et al.* (2018) scATAC-seq data set took approximately 1 hour to run across 8 cores and the matched scRNA-seq data set took approximately the same time across four cores.

Running ProjectR between the Schep *et al.* (2017) data set and the Buenrostro *et al.* (2018) data took less than 15 s on a laptop with an Intel 7th gen i5 processor.

## RESULTS

### The scATAC-CoGAPS algorithm

CoGAPS is a Bayesian matrix factorization algorithm which decomposes a matrix of sequencing data into two output matrices, representing learned latent patterns across all the samples and genomic features of the input data (12,13). The first of these is called the Amplitude matrix, and it contains a numerical representation of the degree to which each feature contributes to each latent pattern learned by the algorithm. The second is termed the Pattern matrix, which represents the degree to which each learned latent pattern is present in each single cell (Figure 1A) (30). Latent patterns are intended to capture common accessibility across both genomic features and cells, and thus identify the regulatory biology common among cells in the data (hereafter they will be referred to simply as patterns). The scATAC-CoGAPS algorithm takes as input a count matrix with reads aggregated across any relevant summary feature (e.g. peak regions or DNA motifs that identify TF binding sites).

The values of the Pattern matrix can be used to distinguish cell types or cell populations specific to each chromatin-accessibility derived pattern. This correspondence allows us to annotate patterns as associated with a particular group of cells. In contrast to standard clustering methods, the patterns learned from CoGAPS can simultaneously identify patterns that delineate individual cell types as well those shared across cell types.

The pattern identified by each row of the Pattern matrix corresponds to a set of gene weights in each column of the Amplitude matrix. These weights provide information on which specific features (peaks, motifs etc.) contribute the most to each pattern. In this way, features can be linked to the cell types or cellular states defined by associated patterns, which enables the identification of the active regulatory programs within each group of cells. Further, these learned patterns can be input to our projectR transfer learning method (14,15) to query their occurrence in related cells in other scATAC-seq datasets.

Assessment of regulatory programs from the amplitude matrix of scATAC-CoGAPS depends upon the features selected for summarization of the scATAC-seq data. The approach outlined here focuses on the annotation of both peaks and DNA motifs. When using open chromatin peaks to define our feature set, we employ two main analysis steps (Figure 1B). First, we match peaks to genes that fall within the regions they cover or have promoters within these regions. These sets of genes can then be compared to known pathways via gene overlap analysis (21), returning significantly overlapping pathways. Peaks can also be searched for known DNA motifs and their possible TF bindings. The frequency of these potential TF binding sites can inform an understanding of which regulatory effectors are characteristic of a specific cell population. While other analysis methods require one particular mode of feature summarization, CoGAPS allows for the use of any feature that facilitates aggregation of reads into a count matrix. If we instead use a feature space initially defined by DNA motifs, we can again match pattern-defining motifs directly to known TF binding sites to determine enrichment for particular TFs, often extending the number of unique regulatory patterns we are able to uncover from the data (compared to using a peak based feature space alone). However, given that a feature space of peaks provides more options to interrogate regulatory biology (i.e. pathways and TF binding versus TF binding alone), we employ peak summarization as default in our analysis throughout, and utilize a motif-defined feature space to supplement this analysis.

### scATAC-CoGAPS differentiates known cell identities in scATAC-seq data

**Figure 2.**
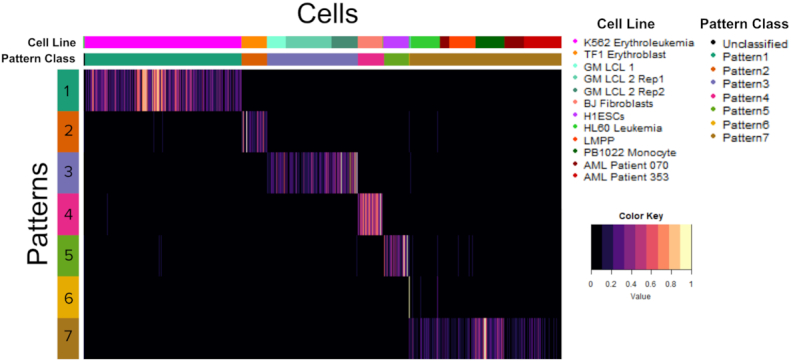
Heatmap of the pattern matrix with cells matched to learned patterns. The color gradient of the heatmap reflects the pattern matrix weights for each cell for each pattern, which indicates the degree to which each pattern is found in each cell, as learned by CoGAPS. Cells are labeled by both Pattern Marker pattern assignment as well as known cell line and culture of origin. Patterns 1–5 all very sharply distinguish a particular cell line. Pattern 6 only captures one cell. Most of the remaining cells are assigned to pattern 7, leaving only five cells without a pattern assignment.

To demonstrate the capacity of CoGAPS to distinguish cell populations, we run the algorithm on publicly available scATAC-seq data published by Schep *et al.* (7). These data derive from 12 cell cultures, comprising ten different known cell lines (listed in Supplemental Table S1). The cell lines in the data are generally well-characterized, which allows for validation of the cell-type specific regulatory programs predicted by scATAC-CoGAPS. Using peaks to define our feature space, we apply CoGAPS to search for seven patterns of accessibility in the data (see Methods for dimensionality selection). After the factorization, we associate each cell with a single pattern using the PatternMarker statistic included in the CoGAPS package (13). Pattern assignments learned by CoGAPS on this data set align well with *a priori* knowledge of cell line annotations (Figure 2, Supplemental Table S2). Cells belonging to the same cell line are almost always assigned to the same pattern (Adjusted Rand Index of 0.90).

Pattern 1 and Pattern 2 perfectly categorize K562 Erythroleukemia and TF1 Erythroblast cells, respectively. GM B-cell derived LCLs, BJ Fibroblasts and H1 Embryonic Stem Cells each have three or fewer cells incorrectly assigned to patterns 3, 4 and 5. We note that Pattern 3 captures all three cultures of GM lymphoblastoid cell lines (GM LCLs), indicating that CoGAPS is differentiating these cell lines via regulatory differences of biology rather than through technical artifacts of cell culture. Pattern 6 is most significantly associated with HL60 Leukemia cells, however, due to the low signal in pattern 6, the patternMarker statistic only assigns one HL60 cell to that pattern, and the rest to pattern 7. Pattern 7 is assigned most of the remaining cells in the data, and while it is most significantly associated with PB1022 Monocytes, it also has significant signal across HL60 Leukemia cells, Lymphoid-Primed Multipotent Progenitors, and the two AML patient cell lines. We hypothesize that the regulatory similarity derived from the shared hematopoietic origin of these cells is responsible for this common signal.

While the CoGAPS solution described above is for seven patterns, the selection of an optimal dimensionality for unsupervised learning remains an open question, and there probably is no single correct number of patterns to use (31). Different numbers of patterns will provide somewhat different information, with more patterns producing more (and usually smaller) groups of cells. Therefore, we also run CoGAPS to analyze the scATAC-seq data for additional dimensions. When increasing dimensionality beyond 7, CoGAPS finds patterns that more strongly differentiate Monocytes and Lymphoid-Primed Multipotent Progenitor cells, but still does not return patterns distinguishing the two Acute Myeloid Leukemia patient cell lines apart from Lymphoid Primed Multipotent Progenitors (Supplemental Figure S1). For example, at the 13-pattern dimensionality, we observe that pattern 1 mainly distinguishes monocytes, while pattern 10 now captures the unifying signal across HL60, LMPP, and AML patient cells. This result indicates that higher dimensionalities allow CoGAPS to identify finer differences between cell types with similar accessibility signatures. At the same time, with this higher dimensionality, patterns 4, 6, 8, 11 and 13 have signal that identifies only single cells. Thus, we observe a tradeoff at higher dimensions between improved differentiation of cell types and an increased number of single-cell patterns, which seem less likely to contain relevant biological signal. Based on our results across dimensions, we retain the seven-pattern solution for our remaining analyses in order to optimize cell type differentiation while minimizing the number of patterns that are associated with only a single cell.

### Analysis of accessible features predicts regulatory programs consistent with established biology of cell lines

**Figure 3.**
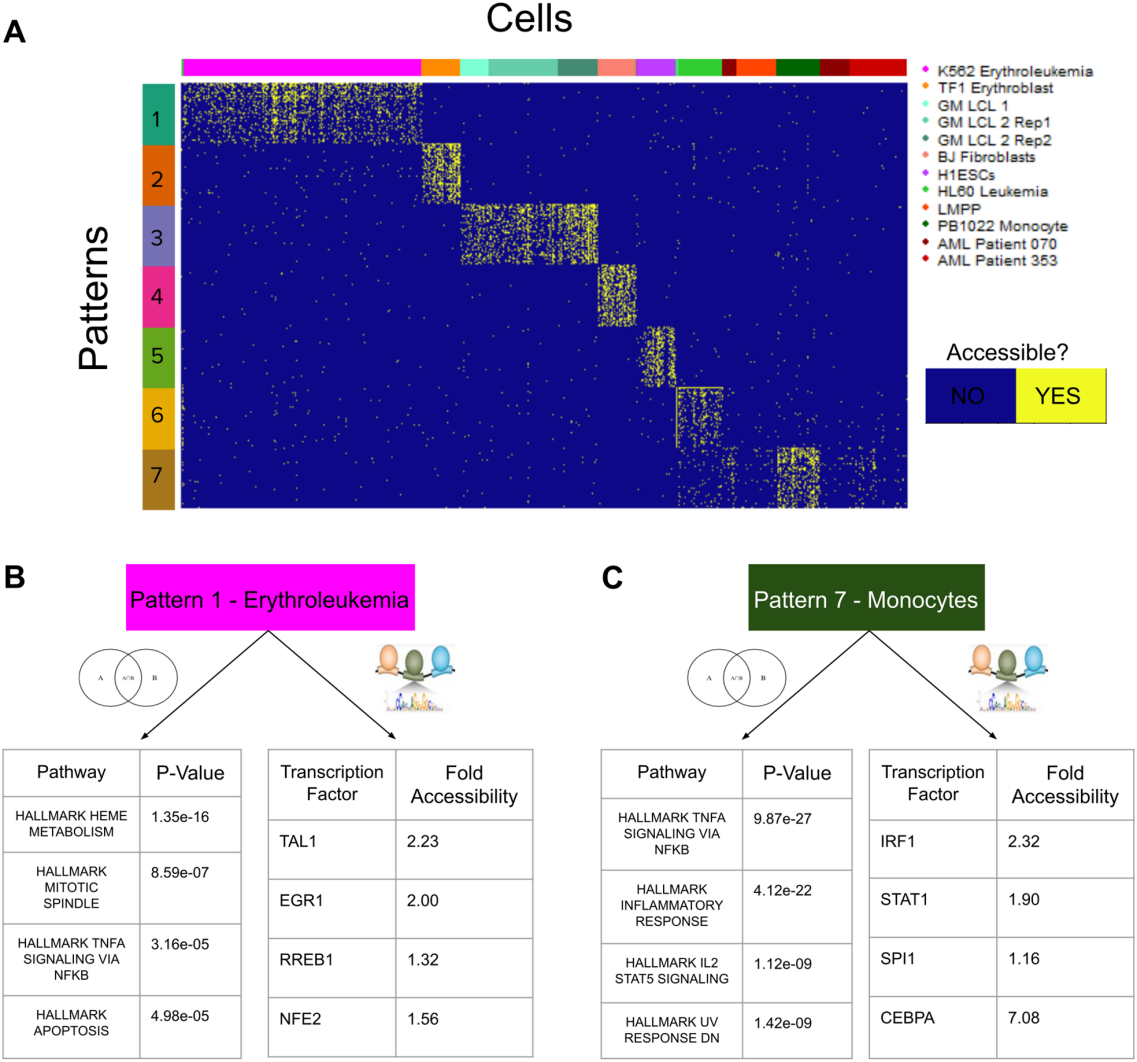
(**A**) The PatternMarker statistic is used to find the 50 most pattern-distinguishing peaks for each pattern. The counts recorded at these peaks from the scATAC-seq experiment are binarized for accessibility and plotted across all cells in the data. (**B, C**) Examples of the MSigDB Hallmark Pathways with significant overlap to genes matched to PatternMarker peaks (the four most significant pathways for each pattern) and Transcription Factors with high numbers of possible binding sites in PatternMarker peaks. TFs listed are those that are both within the top 15 list of TFs with the most enriched binding sites and have highly plausible functional annotations for activity in these cell lines. Fold accessibility refers to the peaks overlapping with the region of the TF gene, relative to other peaks in the K562 Erythroleukemia cell line and PB1022 Monocyte cell line, respectively.

After using CoGAPS patterns from the seven-dimensional solution to define cellular populations, we use the values of the corresponding feature weights in the Amplitude matrix to ascertain which peaks contribute the most to each learned pattern using the PatternMarker statistic. The peaks identified by the PatternMarker statistic reveal the accessible features of the data that themselves strongly distinguish cell types, which we shall refer to as PatternMarker peaks (Figure 3A). For most cell lines, the accessibility of the PatternMarker peaks learned from CoGAPS analysis better distinguishes the cell lines than the pattern weights themselves. This result suggests that the features CoGAPS learns reflect biologically relevant differences in accessibility between the cell populations that it is stratifying. Due to its increased granularity, this analysis provides further evidence that Pattern 6 is characteristic of HL60 Leukemia cells, and that the peaks associated with Pattern 7 are the most accessible in PB1022 Monocytes. At the same time, we observe that the PatternMarker peaks for Pattern 7 have enriched accessibility among other cell lines that were associated to the pattern previously, as compared to cell lines that were not associated. This result indicates that while Pattern 7 most strongly identifies the accessibility signature of Monocytes, CoGAPS identifies the similarities in this signature for other cell types at this dimensionality.

The learned PatternMarker peaks can be associated with cell-specific regulatory mechanisms using pathway and transcription factor enrichment analysis (Supplemental Files 1 and 2). For example, Pattern 1 (the K562 Erythroleukemia-associated pattern) identifies the MSigDB HALLMARK HEME METABOLISM pathway as the most significantly associated with the cell line (Figure 3B). This matches our biological expectation, as increased accessibility of or near genes associated with Heme metabolism is consistent with the erythroid lineage K562 cells derive from. The second most significant pathway is HALLMARK MITOTIC SPINDLE, which suggests the uncontrolled division of this cancer cell line may be driven by epigenetic changes.

Motif analysis from these accessible peaks further identifies TFs with the most accessible binding sites as potentially active regulators in the pattern-associated cell population. The top 15 TFs enriched within the K562 cell associated pattern include TAL1, EGR1, RREB1 and NFE2 which have all been associated with leukemia (32–34) or, in the case of NFE2, is an erythroid nuclear factor. TAL1 is a noteworthy hit, as K562 cells were used to establish TAL1 as a driver of leukemia (32–34), thus providing support for the validity of this approach. To measure the likelihood that the TFs are themselves expressed, we then find the relative accessibility signal at the peaks overlapping the genes of these candidate TFs. All of the above TFs identified from motif analysis also have increased gene accessibility compared to the average peak accessibility in K562 cells, with TAL1 having the highest relative accessibility (Supplementary Figure S2). The accessibility of the gene is most notable for the peak overlapping with the transcriptional start site (TSS) of the gene, with the frequency of the accessibility signal decreasing among the peaks further from the TSS.

The genes overlapping with the peaks that contribute most strongly to the Monocyte-associated Pattern 7 are enriched for the MSigDB HALLMARK INFLAMMATORY RESPONSE and HALLMARK TNFA SIGNALING VIA NFKB pathways (Figure 3C). Both pathways are biologically consistent with the known role of monocytes in immunity and inflammation, as well as with the immunological roles of the other hematopoietic lineage cells secondarily associated with Pattern 7. Within the top 15 TFs with the most enriched binding sites, IRF1, STAT1, CEBPA and SPI1 all have previously established roles in the regulation of monocytes (35–38) and all TF genes have increased gene accessibility relative to average for monocyte peaks in the data (Figure 3C). The pathway and TF enrichment results for all other patterns are listed in Supplemental Files 1 and 2 and provided as R objects at https://github.com/rossinerbe/ATACCoGAPS-Analysis-Code/tree/master/data. Taken together, these results demonstrate the capacity of scATAC-CoGAPS to identify regulatory features of biological relevance from scATAC-seq data.

To examine whether the genomic region annotation-based method we use to determine the relevance of genomic peaks for pathway enrichment produces similar results to GREAT (27), which is a standard tool for determining the functional relevance of genomic elements, we apply GREAT to find genes associated with the peaks learned by CoGAPS from the Schep *et al.* (2017) data set. We then perform the same gene set overlap test to find enrichment of MSigDB Hallmark Pathways for both the significant GREAT gene list as we performed for the gene list generated from our region annotation-based method. The statistically significant pathways from this GREAT analysis are listed in Supplemental File 3 (compare to Supplemental File 1 from CoGAPS) and in easily parsed format at https://github.com/rossinerbe/ATACCoGAPS-Analysis-Code/tree/master/data. We find that GREAT-identified genes produce highly similar Pathway enrichment results. While the GREAT enrichment produces fewer significant pathways, the most significantly associated pathway for each pattern is the same for all patterns (excepting the two patterns for which GREAT analysis returns no significant results).

### Summarization of the count matrix by DNA motifs extends the regulatory patterns CoGAPS learns from scATAC-seq

While using peaks as summarization of ATAC-seq reads provides more avenues for downstream analysis, it has been previously shown that motif-level summarization is an additional information rich feature space for scATAC-seq analysis (7). Therefore, we compare our previous peak-level CoGAPS analyses for the Schep et al. data set to motif-based CoGAPS analyses (labeled Pattern Defining Motifs in Figure 1B) of the same dataset to assess the impact of feature selection on the inferred regulatory programs. CoGAPS analysis of this motif-based count matrix identified 10 total patterns from the data (Supplemental Figure S3A). Patterns 4, 6 and 8 from this motif-level CoGAPS run differentiate GM-LCLs, BJ Fibroblasts and TF1 Erythroblasts, respectively.

The other patterns identify additional cell populations that are not found when the data are analyzed using peak feature space (Supplemental Figure S3A). For example, Pattern 10 identifies regulatory similarity between K562 Erythroleukemia cells and TF1 Erythroblasts, a pattern that peak based analysis does not find (Supplemental Figure S3B). In Pattern 10, we identify high enrichment of candidate TF binding sites for GATA transcription factors, which are known to have critical roles in erythroid differentiation and are shared between Erythroleukemia and Erythroblasts (39). We additionally find that the PatternMarker motifs identified by CoGAPS in this analysis are nearly all different than the motifs found by peak-based analysis. When patterns that seem to differentiate the same cell types are compared, <10% of the motifs identified by each analysis overlap (overlap for Fibroblast associated patterns is given in Supplemental File 4 and provided as an R object at https://github.com/rossinerbe/ATACCoGAPS-Analysis-Code/tree/master/data).

These results suggest that using DNA motif-based summarizations identifies additional regulatory information from the same cell types contained within the same data, and directly supports the use of both peak and motif based summarizations to fully characterize the regulatory biology of cellular subpopulations in scATAC-seq data. Notably, motif-based summarization appears to better identify patterns of accessibility that are shared across multiple cell types, while peak-based summarization better differentiates individual cell types.

### Transfer learning with projectR establishes the generality of the regulatory programs CoGAPS patterns capture

Once we have established signatures of accessibility for cell populations in our data, we employ transfer learning with the R/Bioconductor package projectR (14,15) to determine whether these signatures appear in similar cell populations from other experiments. Notably projectR can efficiently detect the presence of previously learned patterns of accessibility in separate scATAC-seq data as a means of *in silico* validation and discovery. This capability allows for the development of cell population-specific accessibility signatures based on CoGAPS results, which can be used to test for regulatory programs of interest in novel samples.

**Figure 4.**
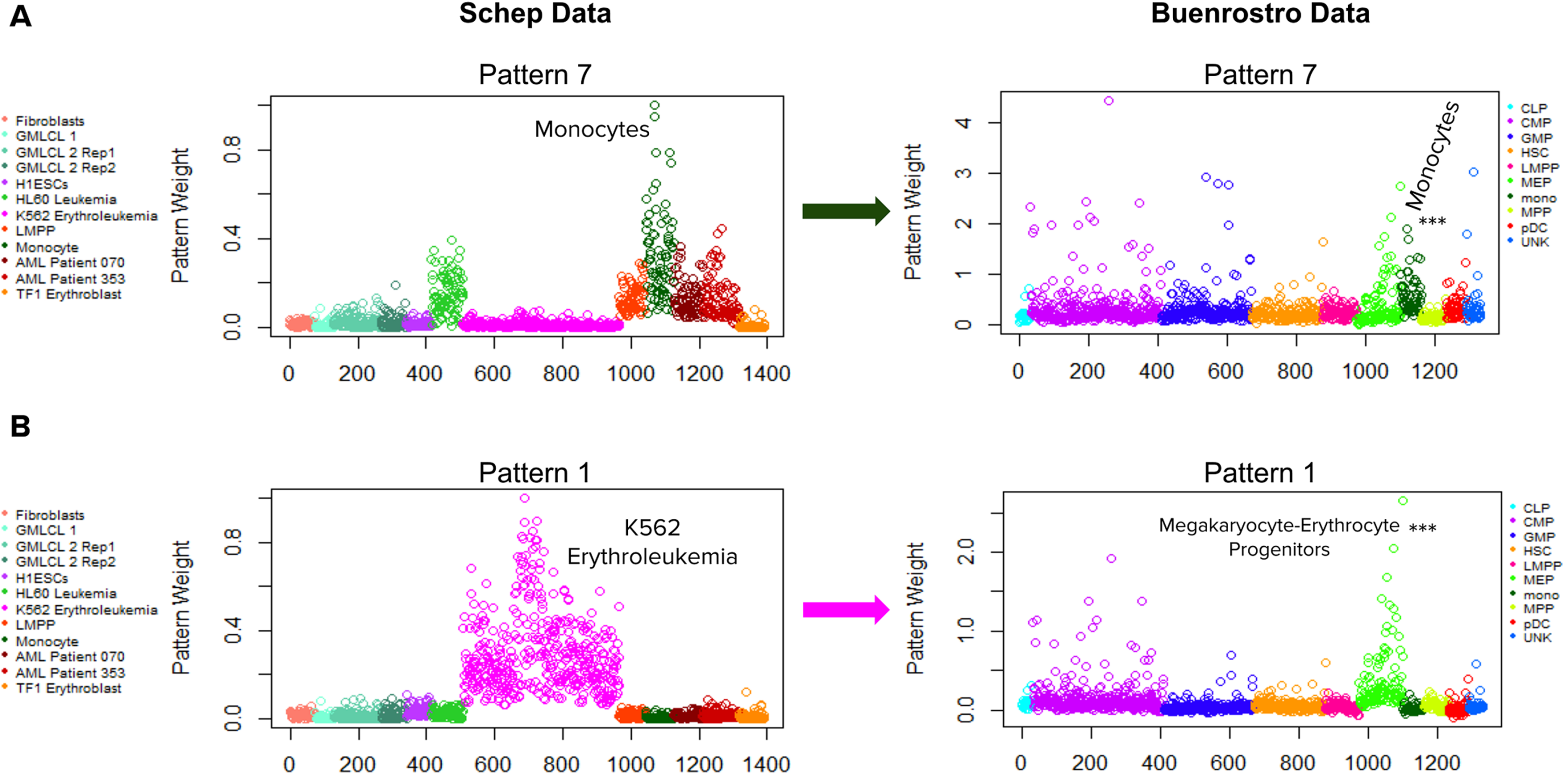
(**A**) Projection of peak accessibility associated primarily with monocytes in the Schep data set into the Hematopoietic lineage Buenrostro data set. The Monocytes in the Buenrostro set are the cell type most significantly associated with the pattern, as determined by a pairwise Wilcoxon Rank Sum Test. (**B**) Projection of the accessibility signature associated with the K562 Erythroleukemia cell line in the Schep data into the hematopoietic lineage data. This signature is most significantly associated with Megakaryocyte-Erythrocyte Progenitor cells.

We demonstrate projectR’s application to scATAC-seq by transferring the patterns learned in peak-level summaries of the Schep *et al.* (7) cell line data to scATAC-seq data from Buenrostro *et al.* (28), which contains 10 different hematopoietic lineage cell types labelled via Fluorescence Activated Cell Sorting (cell types listed in Supplemental Table S3). We project the monocyte-associated pattern (Pattern 7) from the Schep *et al.* data onto the Buenrostro *et al.* data and observe that the monocytes in the target data are most significantly associated to the accessibility pattern (Figure 4A). Comparing average cell line association with the pattern in the target data may make the specificity of the monocyte association more visually clear (Supplementary Figure S4). As previously noted, there is considerable Pattern 7 signal among other non-monocyte hematopoietic-lineage cells within the Schep *et al.* data set, and this is reflected in the general signal observed in the Buenrostro target data set.

ProjectR can also provide information on the regulatory overlap between different cell types. In this case, it provides insight into the regulatory similarity between two distinct cell populations. For example, projection of the K562 Erythroleukemia cell line pattern from the Schep *et al.* data (Pattern 1) into the Buenrostro et al. data has the strongest signal in Megakaryocyte–Erythrocyte progenitors (Figure 4B). This observation supports the presence of overlapping patterns of accessibility between these two populations, consistent with the expected regulatory similarity between Erythroleukemia and Erythrocyte progenitor cells.

### Analysis of matched scRNA-seq data validates regulatory programs learned from scATAC-CoGAPS

When scRNA-seq data is available for cells from the same experimental conditions as scATAC-seq data, we can validate ATAC-CoGAPS predicted TF activity using transcription data of known TF gene targets. CoGAPS analysis is performed as previously described for the scATAC-seq data, allowing for prediction of active TFs. CoGAPS can then be applied to the matched scRNA-seq data to find pattern-defining genes for each cell population as described in (13). These genes can be ranked on the basis of their contribution to each pattern (using the PatternMarker statistic), and then tested for enrichment in the set of genes known to be regulated by a candidate TF using Gene Set Enrichment Analysis (GSEA) (19) (Supplementary Figure S5). In this analysis method, genes known to be regulated by a TF are used as the ‘pathways’ input for GSEA with the ranked PatternMarker genes, including only the TFs predicted to be active by scATAC-seq analysis.

**Figure 5.**
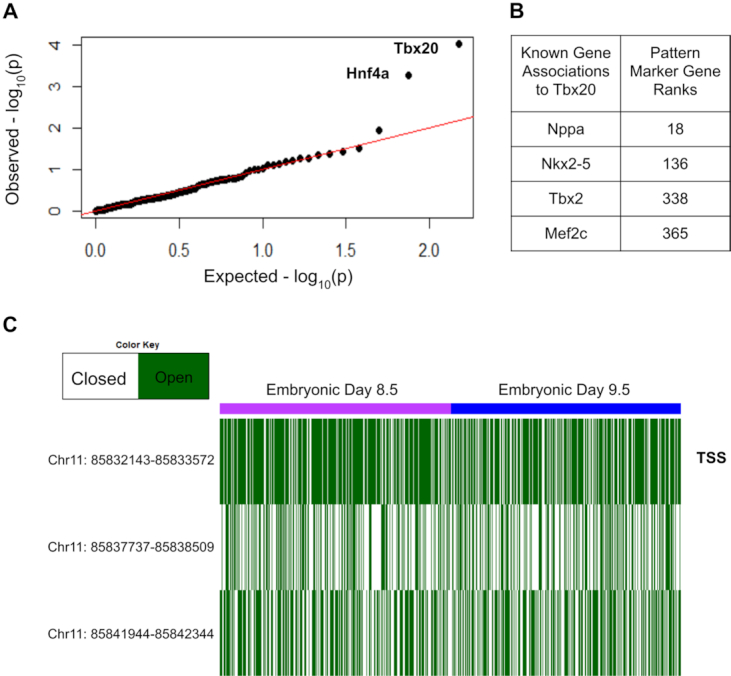
(**A**) qqPlot of *P*-values for gene set enrichment analysis of the Transcription Factors’ gene networks predicted from scATAC-seq CoGAPS and the genes ranked by scRNA-seq CoGAPS. (**B**) Known genes regulated by Tbx20 and their PatternMarker ranks from CoGAPS analysis in matched scRNA-seq. (**C**) Accessibility at the Tbx2 gene in the scATAC-seq data, showing the correspondence of its accessibility and expression levels across mouse cardiac progenitor cells, at embryonic days 8.5 and 9.5. The Transcriptional Start Site overlapping peak (marked with TSS) is the most consistently accessible.

No matching scRNA-seq data was available for the Schep et al. data set. Therefore, we sought to validate this method using matched scRNA-seq and scATAC-seq data from mouse embryonic cardiac progenitor cells at days 8.5 and 9.5 of development, as described by Jia *et al.* (29). We run CoGAPS on both data sets to learn seven patterns in peak-level summarized scATAC-seq data and six patterns in the scRNA-seq data. There is much more regulatory similarity than dissimilarity between cardiac progenitors only one day apart in development, and thus the most distinctive patterns we find in the scATAC-seq data set are those that reflect sustained open chromatin across days 8.5 and 9.5 of development (Patterns 1 and 7) (Supplementary Figure S6). As patterns 3 and 6 from the scRNA-seq experiment also have signal across all cells in the data, we continue by comparing the patterns found across cells rather than the patterns that stratify distinct cell populations. To make this comparison, we first find TFs enriched within the scATACseq data for all cells, and then list the genes known to be regulated by each of the TFs. Then, we find the PatternMarker genes from scRNA-seq from the patterns that show signal across all cell types (patterns 3 and 6). GSEA between the sets of genes regulated by the predicted TFs and the PatternMarker genes provides significant support for Tbx20 TF activity (FDR adjusted *P*-value of 0.015) and Hnf4a activity (FDR adjusted *P*-value of 0.042) across these developing cardiac cells (Figure 5A, B). Tbx20 plays a major role in cardiac development (40), which is consistent with the known biology of embryonic cardiac cells. A homologue of Hnf4a was recently shown to play an important role in normal embryonic development of the chicken heart (41). This result corroborates that finding and suggests that Hnf4a may play a role in cardiac development across a wide phylogenetic range; particularly that it acts in mammals as well.

To investigate the accessibility of genes associated with Tbx20 using scRNA-seq, we find overlapping peaks of said genes within matched scATAC-seq data. The peaks corresponding to the Tbx2 gene and the Nkx2–5 gene are accessible across the cells in the data (fold accessibility 2.39 and 1.51, respectively), while Mef2c and Nppa peaks are less accessible than average (fold accessibility 0.84 and 0.30) (Figure 5C, Supplementary Figure S7). The Tbx2 gene is particularly accessible in the peak overlapping with its transcriptional start site (fold accessibility 3.11). The lack of accessibility among the Mef2c and Nppa genes suggests that accessibility and gene expression do not always align, though we do observe general correspondence between the two data modalities, particularly in transcriptional start site overlapping peaks.

### Comparison to other methods demonstrates the particular efficacy of CoGAPS in identifying biologically relevant patterns

**Figure 6.**
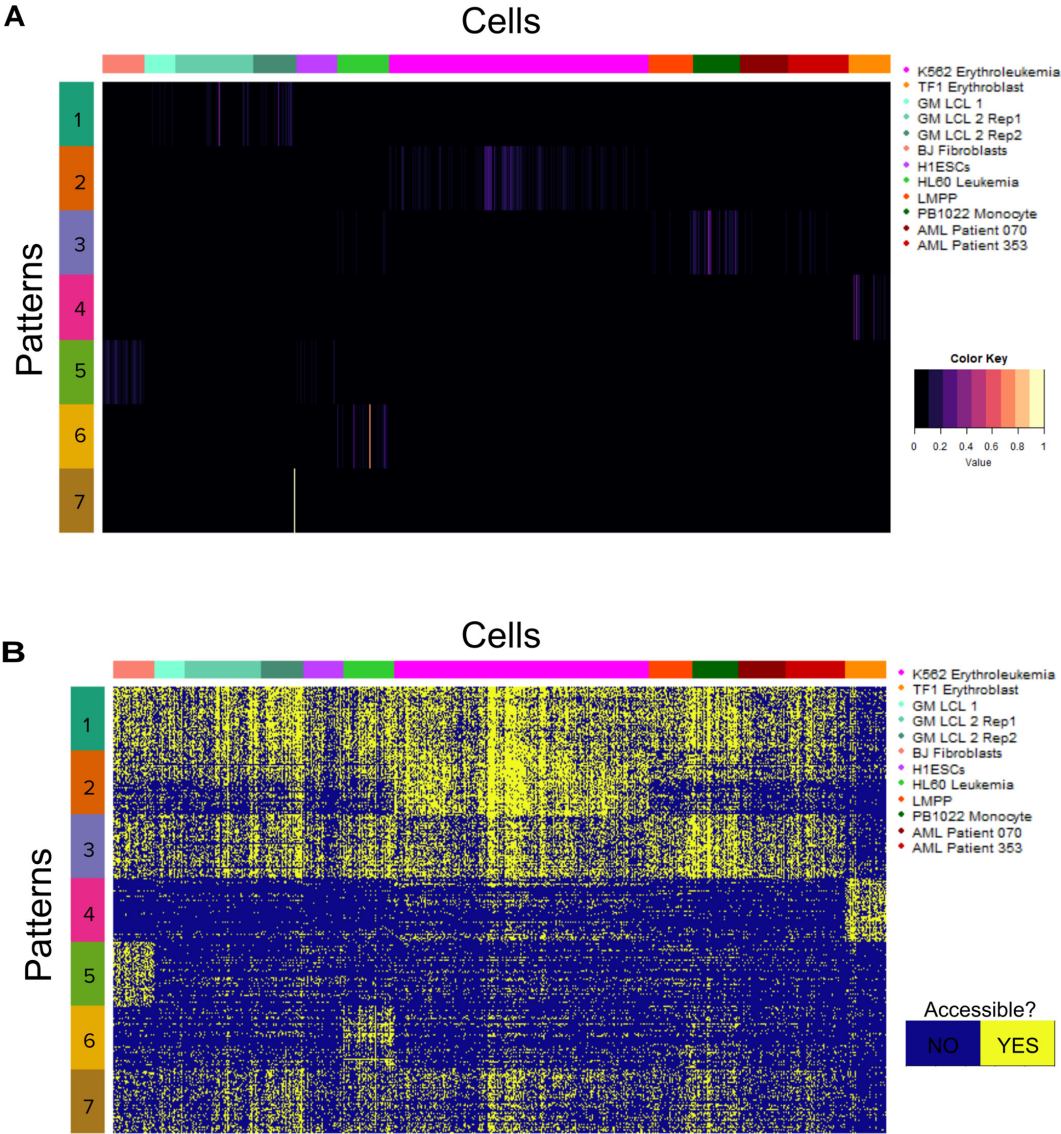
(**A**) Heatmap of the cell weights output by NNDSVD-NMF run on the Schep *et al.* (2017) data. The patterns learned generally distinguish particular cell lines or groups of cell lines together. Intended to provide a comparison with the CoGAPS output plotted in Figure 2. (**B**) Heatmap of the accessibility of the 50 peaks most associated with each pattern from the NNDSVD-NMF analysis. Peaks do not strongly distinguish the cell types the patterns were associated with in (A). Intended for comparison with Figure 3A.

In order to demonstrate the value of applying CoGAPS to scATAC-seq data as opposed to gradient-based Non-Negative Matrix Factorization (NMF) methods, we run the sparse Non-Negative Double Singular Value Decomposition NMF (NNDSVD-NMF) implementation provided by the scikit-learn python library on the Schep *et al.* data set (7), using the coding framework provided by (5). This NMF implementation in nearly as effective as CoGAPS at finding patterns that differentiate the constituent cell lines of the data set (Figure 6A). Pattern 1 identifies cells from the three GM-LCL repeats in one pattern, though it has differentiating signal for less than a third of the individual cells. Pattern 2 differentiates K562 cells, Pattern 3 appears similar to the seventh CoGAPS pattern, showing signal across Monocytes, leukemia, and LMPP cell lines. Pattern 4 distinguishes TF1 Erythroblasts, Pattern 5 has signal for both Fibroblasts and Embryonic Stem Cells, and Pattern 6 has signal for HL60 Leukemia, while Pattern 7 has signal for a single GM-LCL cell.

While this capacity to distinguish cell types is similar to that of CoGAPS (see Figure 2), the features that NNDSVD-NMF associates with these patterns are less specific to cell types than those learned from CoGAPS patterns are (Figure 6B). Only patterns 4 and 5 identify peaks specific to their corresponding cell lines (compare to Figure 3A for the same data plotted from CoGAPS results). Therefore, it appears that while NNDSVD-NMF is able to find patterns that generally distinguish cell types, the biological features learned are less representative of those cell types than those found by CoGAPS.

To further compare our results to a state of the art (42) Bayesian topic modeling method for scATAC-seq analysis, we apply cisTopic (6) to the Schep et al., 2017 data. CisTopic provides a framework for choosing a single dimensionality, which is not available to the NMF algorithms and has a faster run-time than CoGAPS.

**Figure 7.**
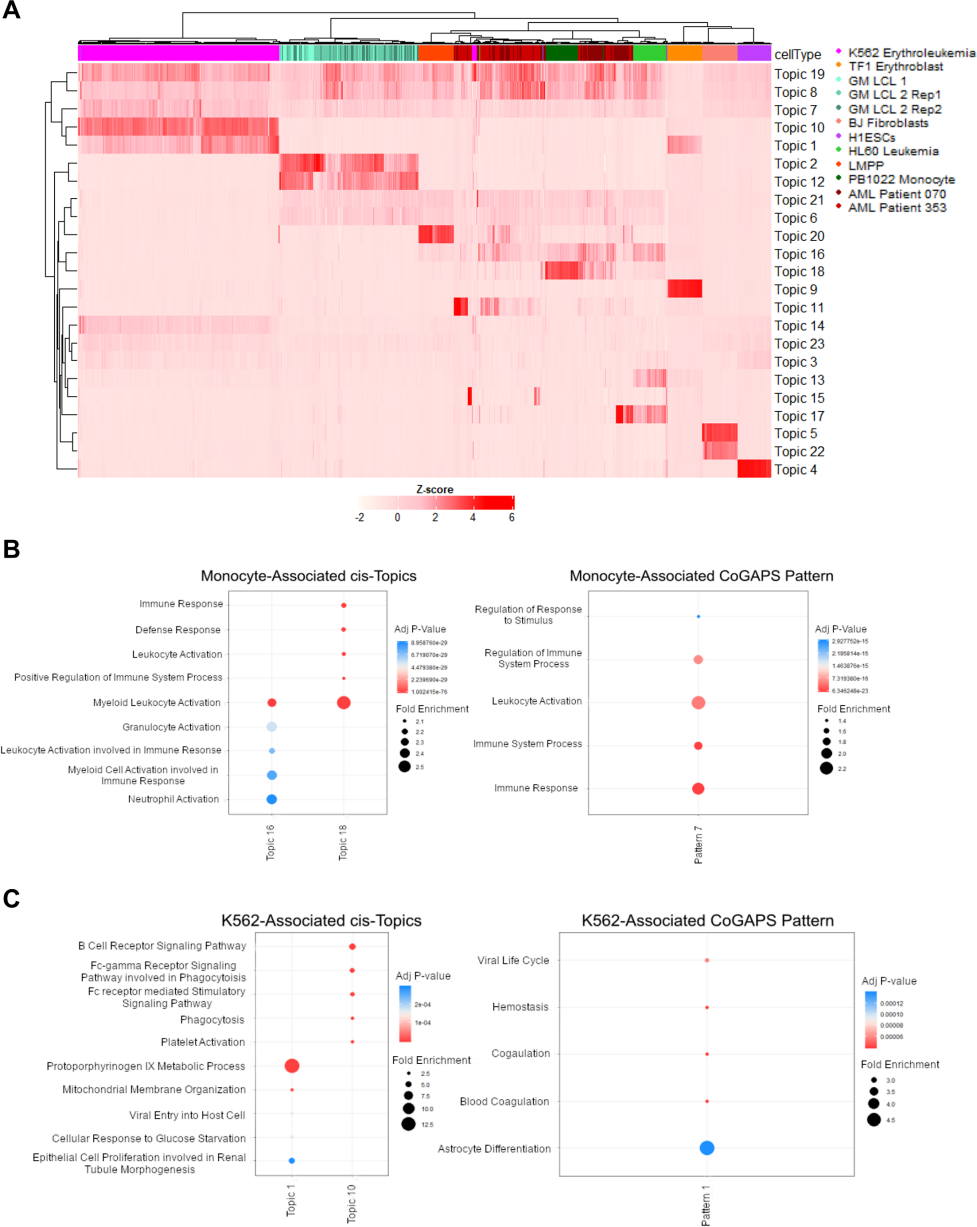
(**A**) Heatmap of cisTopics learned on the Schep et al data set. 23 topics were learned, including topics that differentiate all cell types except the leukemia patients. (**B**) The GO terms significantly enriched for the topics and patterns associated with the PB1022 Monocyte cell line for cisTopic and scATAC-CoGAPS, respectively. Similar GO terms are observed. (**C**) The GO terms significantly enriched for the topics and patterns associated with the K562 Erythroleukemia cell line for cisTopic and scATAC-CoGAPS. The GO terms found diverge considerably.

On the Schep *et al.* data set, cisTopic found 23 regulatory topics in the data set (Figure 7A). cisTopic generally identifies multiple topics per cell type, while CoGAPS more commonly identifies a single pattern per cell type. The topics learned by cisTopic are able to differentiate cell types to a similar degree to CoGAPS. cisTopic is also unable to find latent features which differentiate the two AML patients from LMPP cells or monocytes and, likewise CoGAPS, identifies topics of unifying accessibility between the patient cells, monocytes, HL60 cells, and LMPP cells. These results provide further evidence of the regulatory similarities among these cell lines. cisTopic identifies potentially upregulated pathways based on the peaks that differentiate topics, in a similar manner to what we apply in CoGAPS. Specifically, in the Monocyte associated pattern/topics each algorithm identifies the same or very similar GO terms (Figure 7B): all along the lines of immune regulation, immune response, and leukocyte activation. For these cells, the algorithms appear to be identifying roughly equivalent regulatory systems. However, when we turn to the pathways that have significant association to K562 Erythroleukemia cells, there is considerable divergence. CoGAPS primarily identifies terms associated to hemostasis and blood coagulation (not unsurprising in an Erythroleukemia), while cisTopic finds pathways of phagocytosis and Fc receptor signaling (Figure 7C), which are receptors known to exist on K562 cells (26). Each also returns different pathways that make very little sense in K562 cells: astrocyte differentiation from CoGAPS and protoporphyrinogen IX metabolic process and epithelial cell proliferation involved in renal tubule morphogenesis from cisTopic. It is possible these pathways appear due to the epigenetic dysregulation of this cancer cell line (43). The fact that CoGAPS and cisTopic identify different pathways, each that align with known biology of the cell line, suggests that each algorithm may often find different, legitimate regulatory patterns in scATAC-seq data. Thus, neither method alone is able to capture all relevant regulatory features contained within the data. However, cisTopic does not readily output features that are applicable for transfer learning with ProjectR to enable comparable cross-study and cross-platform data analysis with CoGAPS.

Lastly, to assess our integrated approach for using scATAC-seq and scRNA-seq data to predict TF activity in particular cell types, we compared to the integrative clustering and TF motif enrichment tools provided by the popular single-cell software package Seurat (26). We apply the Seurat integrated workflow to the Jia *et al.* (2018) data set (29) and find that is does not noticeably improve differentiation of the cells in the data (Supplemental Figure S8A) relative to what is produced by CoGAPS patterns (Supplemental Figure S6), with both algorithms appearing to distinguish some minor cell populations, but not along the known axis of day of development. We then tested which TF motifs were found to be enriched in the different populations found by UMAP clustering in Seurat (Supplemental Figure S8B). None of the comparisons identified any cardiac specific TFs, though several general developmental TFs were found. Because of this reliance on clustering, Seurat is limited to comparison of TFs that are enriched between clusters as opposed to TFs shared across multiple features in NMF or topic modeling.

## DISCUSSION

Single-cell epigenomics methods such as scATAC-seq capture a wide array of regulatory features genome wide, but our ability to extract this information is still limited. Here we present the application of CoGAPS and projectR to scATAC-seq, providing an analysis framework for Bayesian Non-Negative Matrix Factorization to uncover regulatory information from sparse, high-dimensional epigenomics data and project these learned patterns across data sets and sequencing platforms.

CoGAPS (Coordinated Gene Expression in Pattern Sets) was originally developed for the analysis of gene expression data. The ability of CoGAPS to extract relevant patterns from different data sources is a great strength of the algorithm. Here, we leverage this capacity to develop a basic framework for integrative analysis of multiple scATAC-seq and scRNA-seq data sets. Since CoGAPS can be applied to any sequencing technology that can produce a count matrix, this framework we present has the potential to support the integrated analysis of additional multi-omics data sets. The importance of this capacity continues to grow with the increasing affordability and concomitant ubiquity of sequencing technologies, and the massive and varied data sets such technologies produce. Furthermore, CoGAPS allows for the summarization of reads to any relevant genomic feature (e.g. peaks, DNA motifs etc.) and facilitates the learning of a wider range of regulatory patterns than methods that require a specific summarization method.

One of the main reasons to apply single-cell analysis methods is to identify heterogeneity in cell populations. This can be particularly useful in the context of identifying differences between diseased cells and healthy cells. The fact that these abnormal cell populations can be quite small can cause problems for dimensionality reduction methods. For many dimensionality reduction techniques, it can be difficult to determine whether the association of a small population of cells with a particular dimension is due to technical or biological factors. The ability of CoGAPS to quantify both how well the cells fit an identified pattern (as shown in Figure 2) and how strongly each genomic feature is associated with the pattern (as shown in Figure 3A) can provide considerable insight into whether a pattern is biological in origin. Patterns 6 and 7 from the peak based analysis of the Schep *et al.* (2017) data set provide an excellent example of how we can clarify the biological meaning of the patterns CoGAPS identifies. When we plot the Pattern matrix in Figure 2, Pattern 6 has notable signal for only a handful of HL60 Leukemia cells. Pattern 7, conversely, has signal across many cells and cell types. Without a good way to observe the contribution of biological features to each pattern, it could be challenging to interpret these two patterns. However, as we see in Figure 3A, when we plot the genomic peaks most associated with each pattern, it becomes much clearer that each pattern is finding something biologically distinctive. Pattern 6 is most associated with peaks that are more accessible in HL60 cells than any other cell type in the data, allowing us to interpret that pattern as an HL60 leukemia pattern. Pattern 7 peaks are much more strongly associated with PB1022 monocytes than any other cell type, suggesting the biology of the pattern is primarily driven by that cell line. This further indicates that the other cell types associated with that pattern have considerable overlap in accessibility signature with monocytes. Still, distinguishing when unsupervised learning methods uncover technical artifacts from biological features without complete *a priori* annotations remains a critical area of future research in single cell analysis methods.

We further extend our ability to validate the biological relevance of CoGAPS patterns through both cross-study and cross-platform analyses with transfer learning from projectR (14,15). The projectR software package makes it possible to determine whether the patterns learned in one data set are present in others, and can do so in a way that is fast and easy to implement. This a major strength of the approach we present, as it helps to simultaneously extend and validate learned regulatory patterns, while also allowing for the comparison of regulatory biology in multiple scATAC-seq data sets. Most current scATAC-seq analysis methods are limited in application to a single data set and any results cannot be directly related to other data sets or analyses. This fact severely limits the efficiency of broad analyses, and the information that can be learned from distinct but complementary data sets. ProjectR thus synergizes with CoGAPS and has tremendous potential for use in analyzing disease-specific data sets. For example, if we can establish robust signatures of disease or treatment associated biology, such as genomic dysregulation and markers of drug efficacy, respectively, we can use CoGAPS and projectR to leverage clinical data for an improved understanding of disease mechanisms (44,45) and to guide treatment decisions.

The projectR transfer learning software is broadly applicable for features learned with unsupervised methods in addition to CoGAPS (15). This flexibility of projectR will support further cross-study analyses with emerging scATAC-seq methods (30). While this study demonstrates the robustness of CoGAPS for inferring regulatory biology from scATAC-seq data, we resolve different aspects of that biology at different dimensionalities and data summarizations. We hypothesize that accounting for these features across hyperparameters as well as additional features informed from ensembles of features learned from alternative methods are critical to resolve the complex landscape of regulatory biology encoded in the data, consistent with emerging literature on multi-resolution methods (46).

We compared cisTopic and CoGAPS to determine how well our analysis framework performed relative to a state-of-the-art Bayesian scATAC-seq analysis method. Each algorithm has some particular advantages: CoGAPS in the capacity to check for the presence of learned regulatory signatures in other datasets using projectR, the ability to use alternative feature summarizations beyond genomic peaks, and application of scRNA-seq CoGAPS for integration of data modalites, while cisTopic provides a more straightforward procedure for choosing dimensionality and faster algorithm runtime. A critical note we find by comparing the results of each on the Schep *et al.* (2017) data is that the two algorithms seem to relatively regularly find non-overlapping latent features of the data, with neither forming a complete picture of the regulatory mechanisms encapsulated in scATAC-seq. This is consistent with the findings that a more complete regulatory landscape is uncovered through analysis of CoGAPS patterns across multiple dimensions. Together, these findings support ensemble-based and multi-resolution methods to completely map the regulatory landscape of biological systems from single cell data consistent with other recent studies of unsupervised learning methods (46).

Another finding from this work that may aid the development of ATAC-seq analysis methods in general is that TF motif-based analysis tends to find more patterns that have signal across cell types, while peak-based analysis finds more cell type specific signal. We hypothesize that each peak mostly contains signal corresponding to one or a few genes, and therefore peaks more finely map cell populations to distinct cell types. Transcription factor motifs, on the other hand, contain signal corresponding to larger regulatory changes that are more likely to be shared between cell types (47), and thus analysis in this space yields more patterns with signal across cell types. If this hypothesis is correct, it seems possible that an enhancer-based space could provide another higher order feature, that could identify more patterns of regulatory biology that act across multiple cell types. Using these different summarization levels, separately as we do in this work, or in an integrated framework, has the potential to improve how well our analyses correspond to the different mechanisms by which genes are regulated.

Matrix factorization is well suited to the problem of understanding scATAC-seq data, as the technique learns patterns that distinguish both features and cells within the two factorized output matrices. This output is conducive to a more thorough analysis of the regulatory differences between the cell populations in the data than most available methods can provide. Thus, it is unsurprising that matrix factorization has been previously applied to scATAC-seq analysis (48–50). We use CoGAPS because it's Bayesian optimization of the factorization finds more biologically distinctive features than standard NMF implementations, as we display with the comparison to NNDSVD-NMF in this work. Duren et al. and Zeng et al. each have applied a coupled factorization for integrative analysis of multiple sequencing modalities, allowing for simultaneous clustering and investigation of regulatory biology (48–50). ProjectR can potentially be applied to the output of these coupled factorizations, allowing for transfer of these integrated patterns of regulatory biology across data sets. Coupled factorization may be a promising avenue for future development of integrative analysis with CoGAPS, and projectR will be able to serve in this context to determine whether different coupled factorization methods identify similar patterns of regulatory biology.

The methodology we introduce to use expression (from RNA-seq) of TF targets to provide orthogonal evidence for the activity of scATAC-predicted TFs was shown to identify TFs associated with known and novel regulatory cardiac developmental biology in a very specific and generally homogenous cell population (days 8.5 and 9.5 of developing cardiac cells in a mouse embryo) (29). The TF motif enrichment method employed by the popular analysis suite Seurat was unable to identify enrichment of cardiac development related motifs in this data. This result seems likely to be largely due to the fact that their method relies on the comparisons of subsets of the data and cannot find motif enrichment across cells, whereas CoGAPS patterns can identify uniform accessibility signal present in the supermajority of cells. Using RNA-seq data to validate TF activity is further recommended from a conceptual perspective. Any ATAC-seq analysis method will not be able to determine if, for example, the TF of interest has suffered a functional knock-out or otherwise is inactivated in a way that leaves its genomic region accessible (51). At the same time, RNA-seq analysis cannot determine if the expression of a set of genes is due to a particular TF if those genes can be regulated by multiple factors. Thus, the use of both lines of evidence provides considerably more confidence as to the important regulatory actors in a biological process of interest – a consideration of particular interest for studies attempting to discern molecular targets for disease treatment.

We note that multi-platform data integration is a broad area of research, extending well beyond matrix factorization-based approaches. Coupled correlation analysis has recently been applied to scATAC-seq and scRNA-seq, both allowing for integrative analysis and imputation of spatial transcriptomics information (26). Linked Self-Organizing Maps have also been used in this context, providing the capacity to find differences between relatively similar cell types (52). In the area of experimental methods development, recent research has provided techniques for parallel sequencing of RNA, accessibility, and methylation from single cells, vastly lowering both the time and monetary cost of joint profiling of single cells (53,54). Further, multiple efforts are underway to sequence transcriptomics and chromatin accessibility from the same single cell, which promises to improve the fidelity of multimodal analysis and the ability of multi-omics computational methods to learn the regulatory biology of constituent cell populations.

## DATA AVAILABILITY

The original ATACCoGAPS software package used to perform analysis of scATAC-seq data in this work is freely available at https://github.com/FertigLab/ATACCoGAPS. All processed input data is available on GitHub at https://github.com/rossinerbe/ATACCoGAPS-Analysis-Code/tree/master/data and also on Figshare at https://doi.org/10.6084/m9.figshare.11959062.v1.

## Supplementary Material

gkaa349_Supplemental_Files
