# Supplementary material for: Matrix factorization and transfer learning uncover regulatory biology across multiple single-cell ATAC-seq data sets
Source: Nucleic Acids Res. 2020 May 11;48(12):e68. doi: 10.1093/nar/gkaa349 (PMC7337516; doi:10.1093/nar/gkaa349)
Supplement: gkaa349_Supplemental_Files [file gkaa349_supplemental_files.zip › SupplementalFiguresNAR.pdf]

Supplemental Figure 1

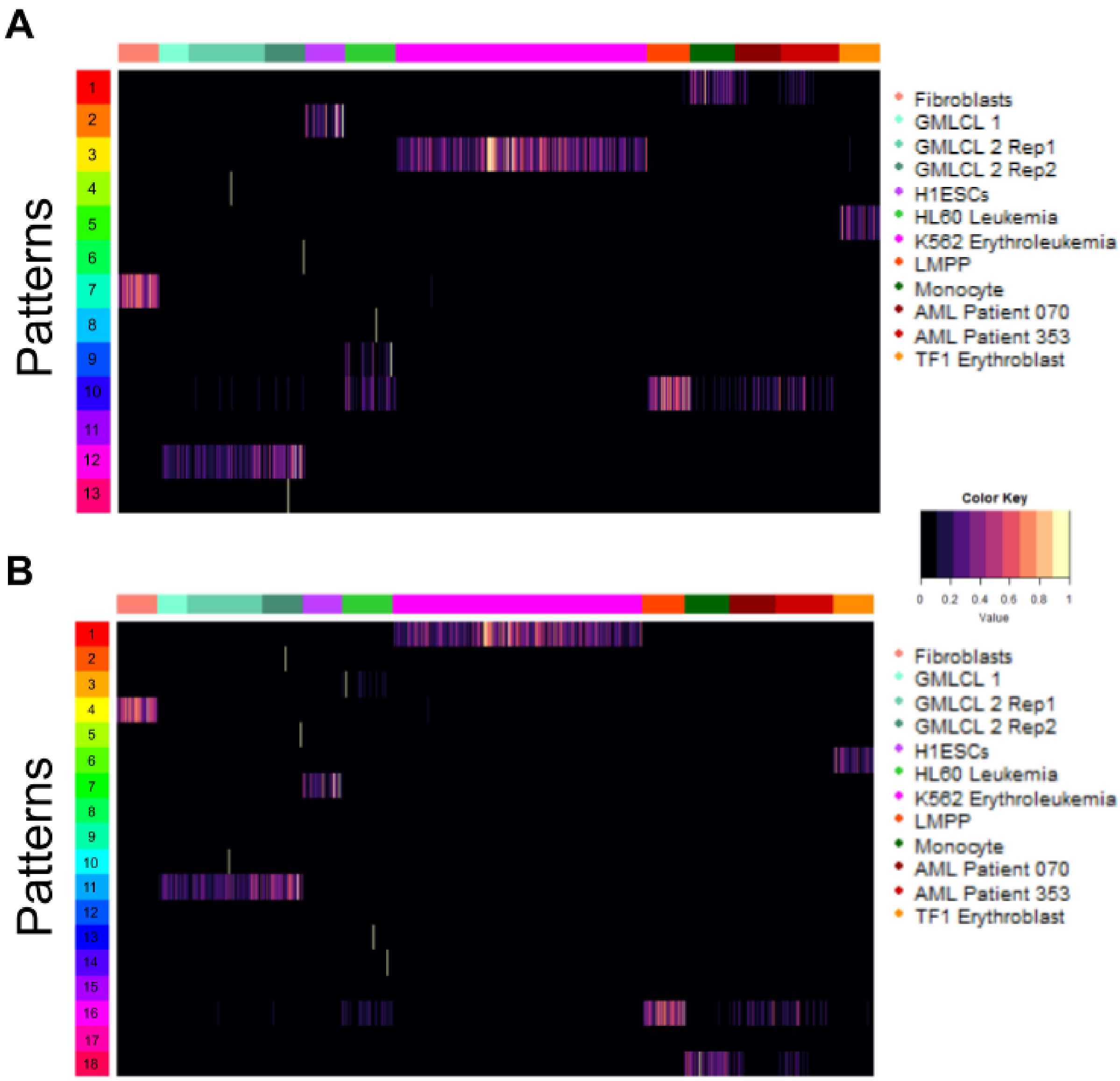

The Pattern matrix is plotted for CoGAPS runs using **A** 13 and **B** 18 patterns for the Schep et al., 2017 data. Several patterns that only have signal for a single cell are observed. The monocyte pattern becomes more clear and a LMPP and patient leukemia pattern emerges, which are not seen when running the algorithm for 7 patterns. Pathway enrichment and TF prediction results are robust across different pattern numbers (i.e. the patterns that distinguish the same cell types return the same most significant pathways and most enriched TFs for

patterns defining the same cell lines) (see Analysis Code).

Supplemental Figure 2

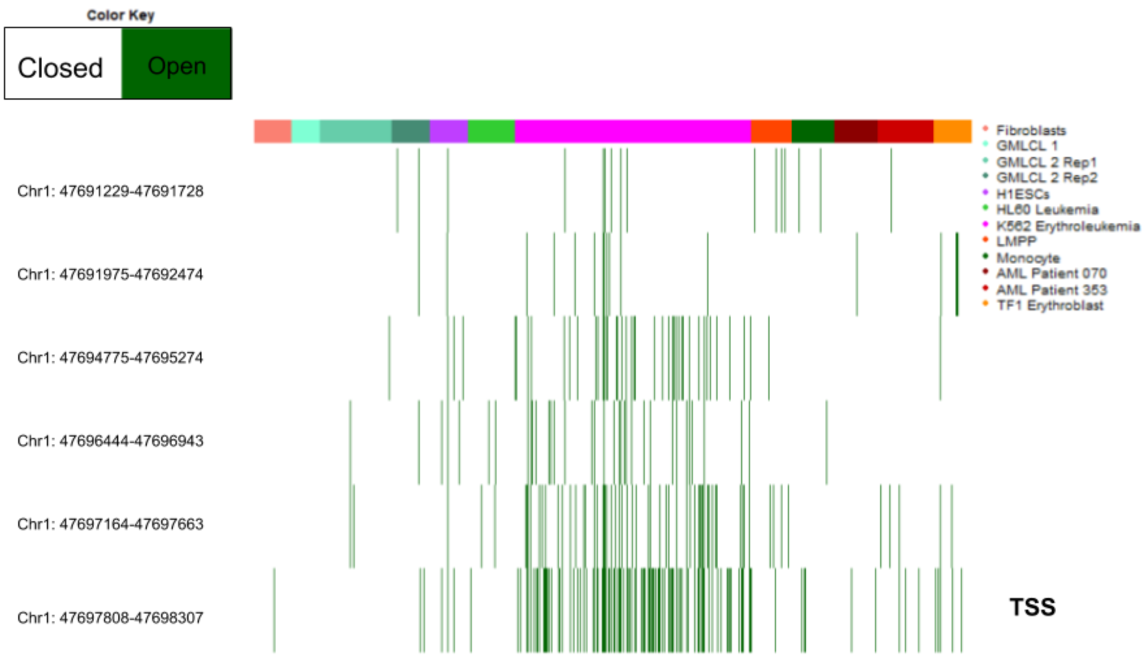

Plot of the binary accessibility of TAL1 overlapping peaks, revealing higher accessibility in K562 Erythroleukemia cells and providing evidence of its specific expression in that cell line. The peak overlapping with the Transcriptional Start Site is marked as TSS and is more consistently accessible among K562 cells than any other TAL1 overlapping peak.

Supplemental Figure 3

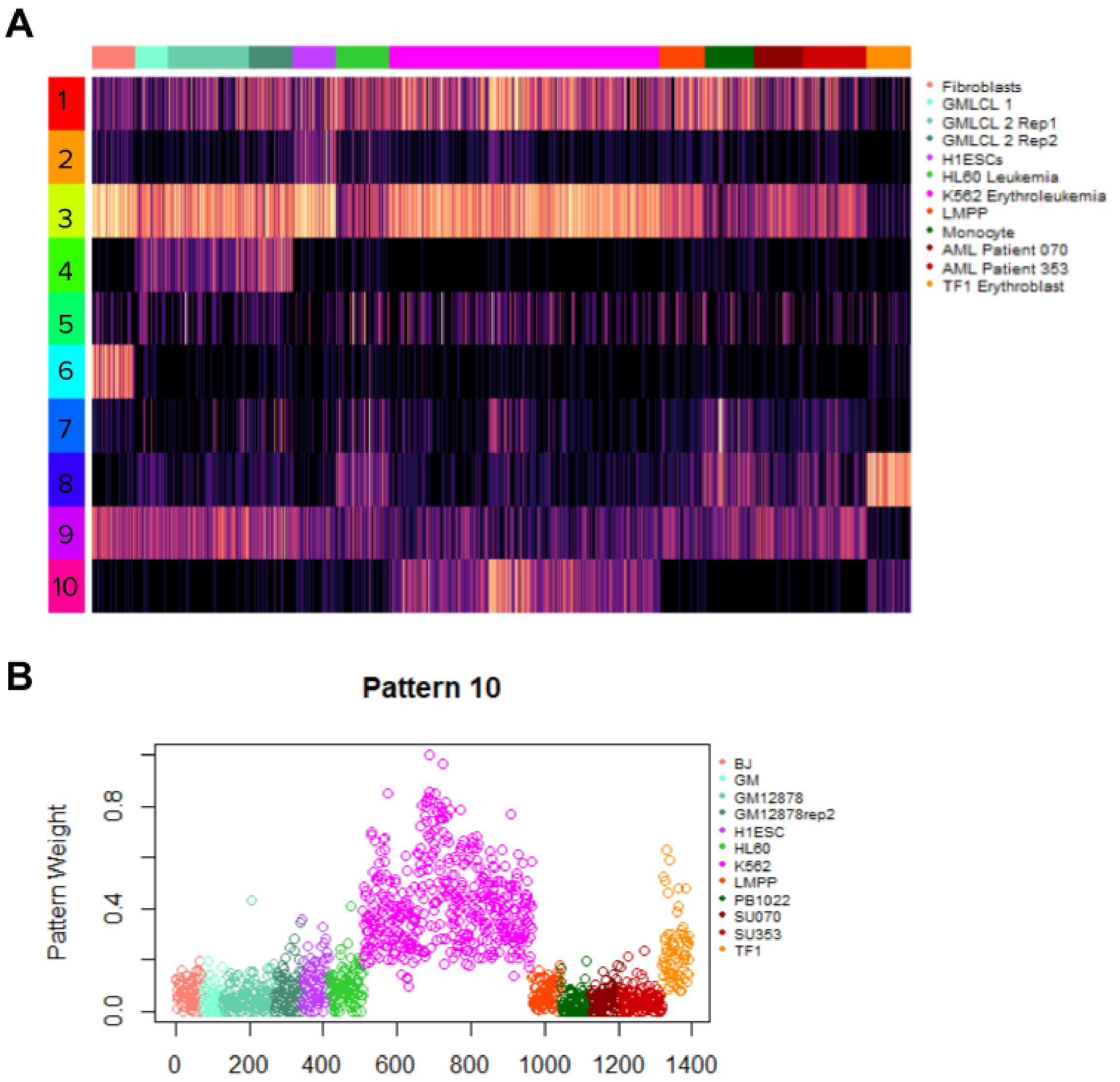

A Plot of the Pattern matrix after running CoGAPS with DNA motif summarization on the Schep et al., 2017 data. The only parameter differences from the peak summarization are that this data is run for 10 patterns and it is run across fewer parallel cores due to there being fewer motifs than peaks. **B** Plot of a pattern found by CoGAPS in the Schep data set when it was run using motif summarization rather than peak summarization (the same as the 10th pattern plotted in **A**, plotted alone for increased visual clarity). Both TF1 erythroblasts and K562 Erythroleukemia cells are strongly associated with this pattern. We do not identify a similar pattern with

summarization to peaks.

Supplemental Figure 4

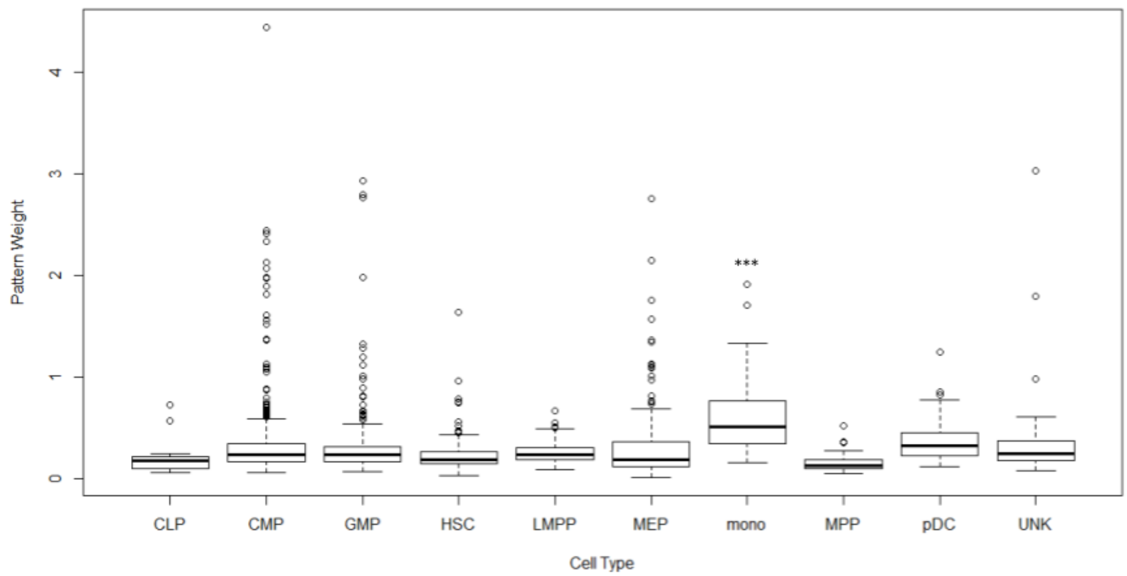

Boxplot of the pattern weight for the transfer of the monocyte associated pattern from the Schep et al. data into the Buenrostro et al. data. The monocytes in the Buenrostro data are most significantly associated with the pattern as evaluated by a Wilcoxon Rank Sum Test.

Supplemental Figure 5

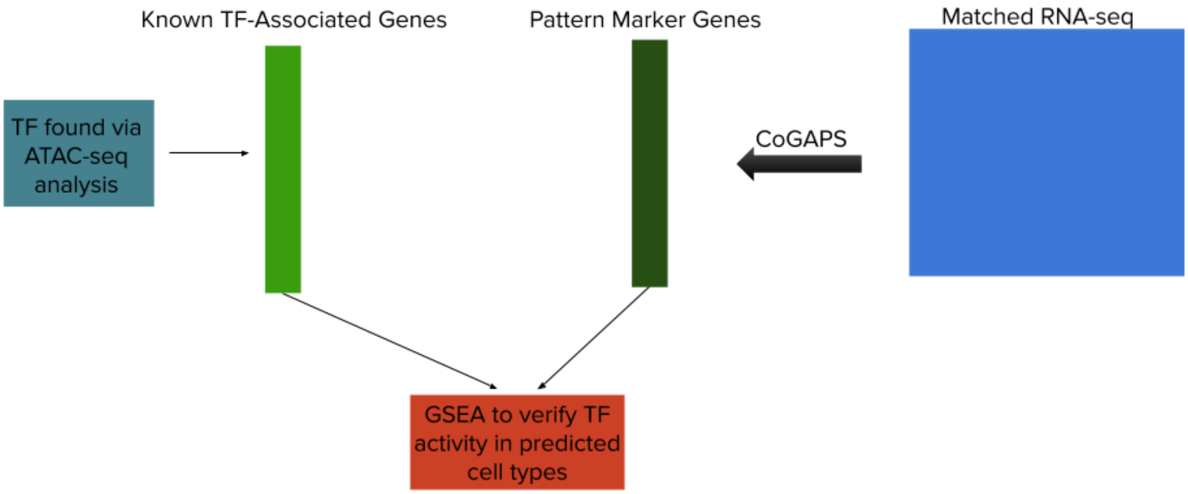

Diagram of the analysis method employed to validate ATAC-CoGAPS candidate using matched scRNA-seq data. The TFs identified by ATAC-CoGAPS are first matched to the sets of genes they regulate. Then, CoGAPS is run on the scRNA-seq data and PatternMarker genes are identified. GSEA is performed between the TF gene sets and the PatternMarker genes to provide transcription-based validation of TF activity.

Supplemental Figure 6

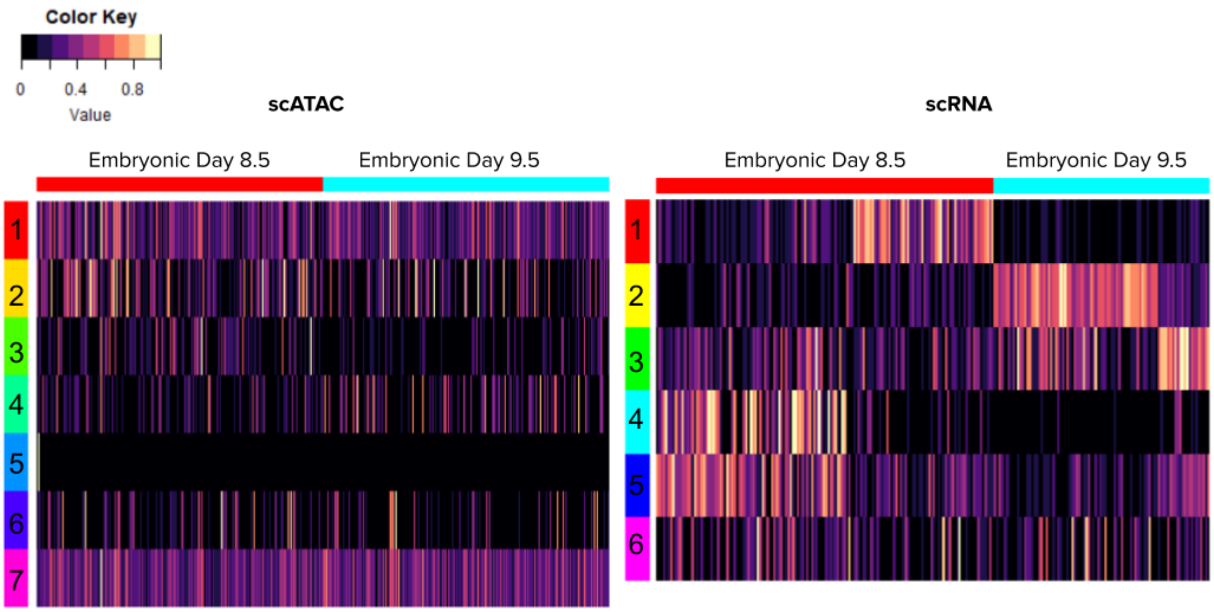

The Pattern matrices plotted for both scRNA-seq and scATAC-seq from matched cardiac development data derived from mouse embryos and published by Jia et al. scRNA CoGAPS finds more differentiating patterns, while most of the scATAC patterns are unifying across the similar cell types, suggesting scRNA-seq is either identifying populations subtypes that scATAC does not capture or is identifying batch effects in the RNA-seq data.

Supplemental Figure 7

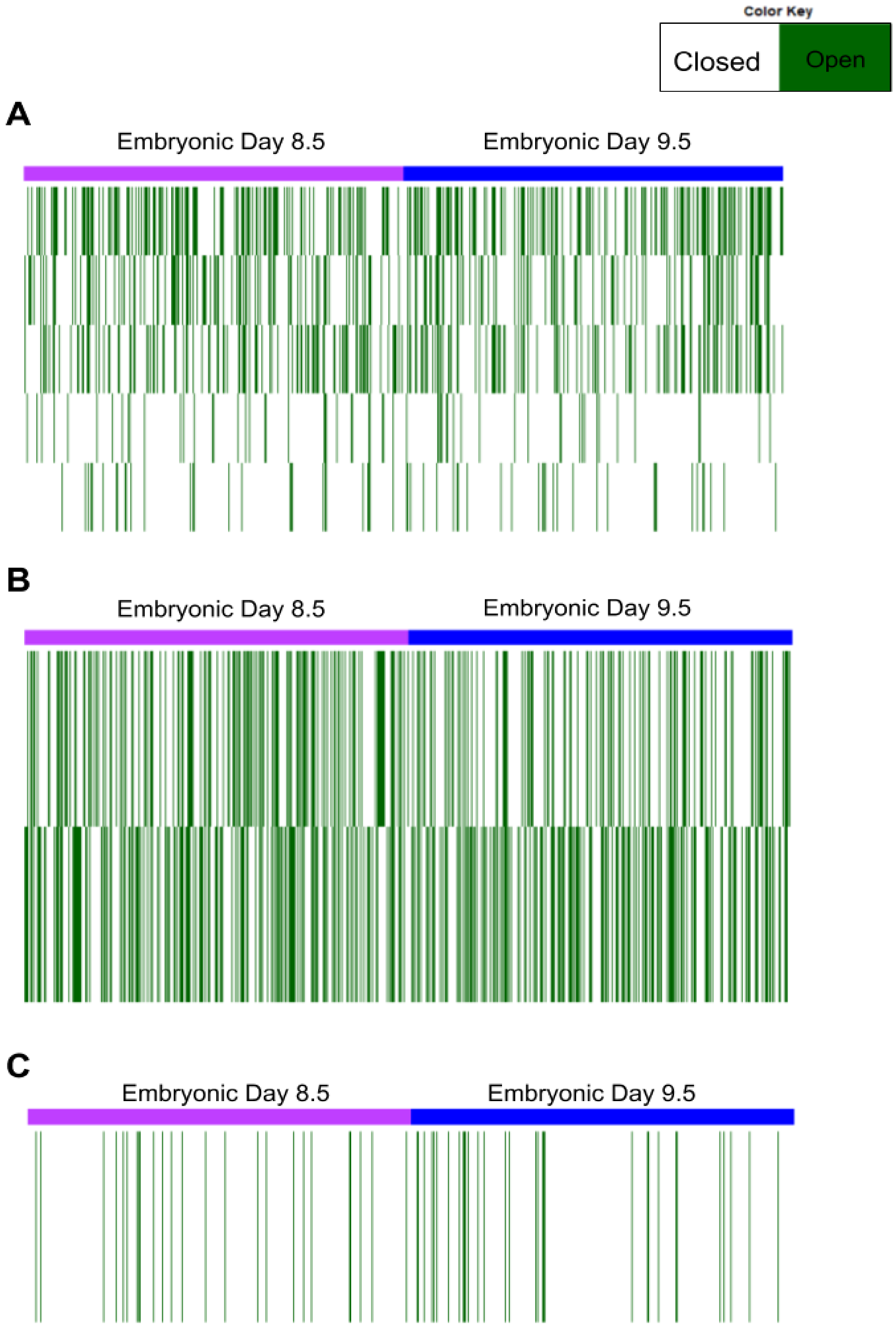

Plot of peaks with overlapping accessibility for the **A** Mef2c, **B** Nkx2-5, and **C** Nppa genes in the Jia et al., 2018 cardiac progenitor data.

Supplemental Figure 8

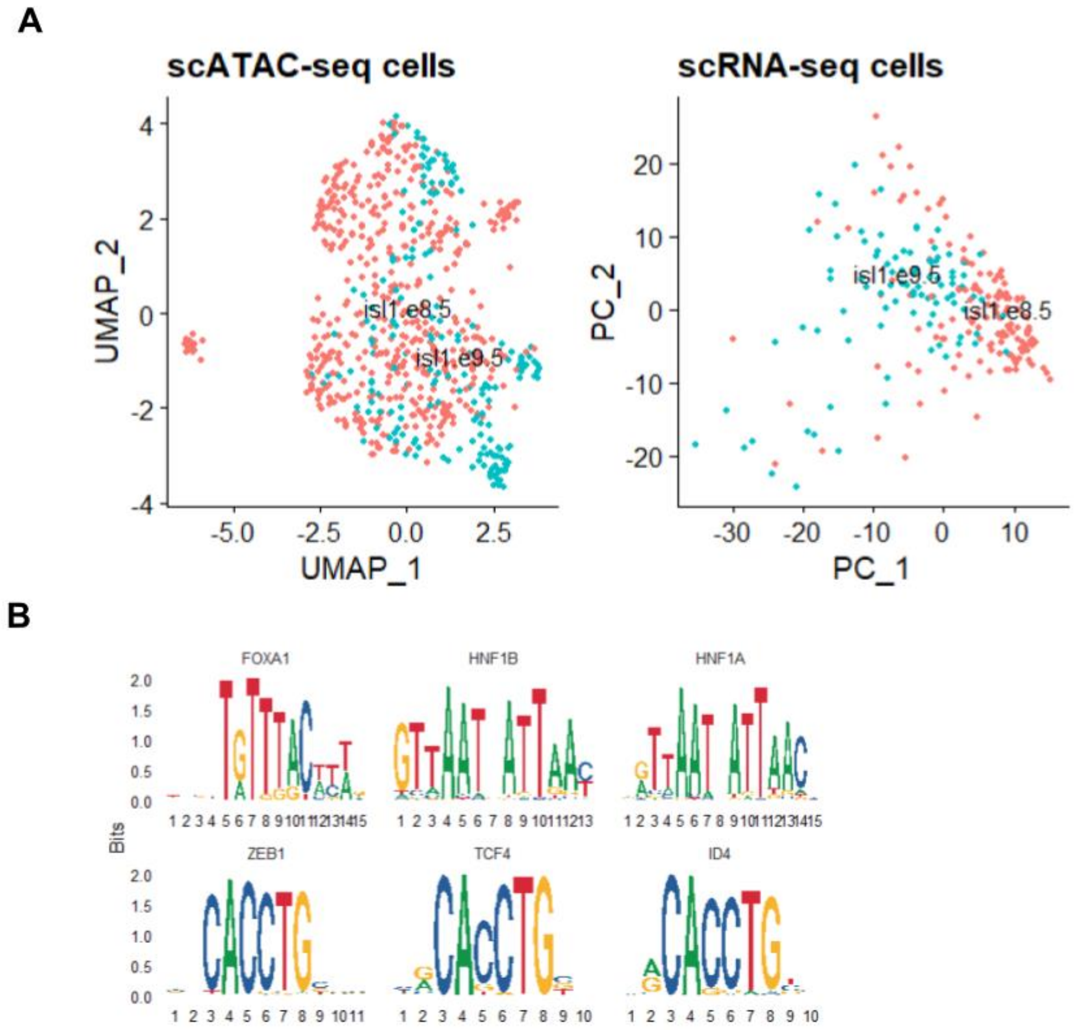

A UMAP of the scATAC-seq data and scRNA-seq data from Jia et al., 2018 after use of Seurat's label transfer and data integration functions. **B** Motif plot of the TF motifs found to be enriched in a comparison of UMAP clusters as determined by Seurat's daughter package, Signac.

Supplemental Tables

Supplemental Table 1

Cell Line	Cell Type
K562	Erythroleukemia
TF1	Erythroblast
GM-LCL	B cell derived Lymphoblastoid cell line
BJ	Foreskin Fibroblast
H1ESC	Embryonic stem cell
HL60	Leukemia (derived from human acute promyelocytic leukemia)
LMPP	Lymphoid Primed Multipotent Progenitor
PB1022	Monocyte
SU070	Acute Myeloid Leukemia, Patient 070
SU353	Acute Myeloid Leukemia, Patient 353

List of the cell lines used in the Schep et al. data set, including the corresponding acronyms used to describe and label them.

Supplemental Table 2

Pattern	Corresponding Cell Line(s)	Cells Assigned to this Pattern	Total Cells	AUC
1	K562 Erythroleukemia	458	458	1.00
2	TF1 Erythroblast	74	74	1.00
3	GM-LCL	266	268	0.996
4	BJ Fibroblast	77	74	0.999
5	H1ESC	74	73	0.999
6	N/A	0	0	N/A
7	HL60, PB1022, LMPP, SU070, SU353	442	445	0.99

Annotations of patterns to cell types and the area under the receiver operating curve for these correspondences based on PatternMarker pattern assignment of each cell.

Supplemental Table 3

Cell Type Abbreviation	Cell Type
CLP	Common Lymphoid Progenitor
CMP	Common Myeloid Progenitor
GMP	Granulocyte-Monocyte Progenitor
HSC	Hematopoietic Stem Cell
LMPP	Lymphoid Multipotent Progenitor
MEP	Megakaryocyte-Erythrocyte Progenitor
mono	Monocyte

MPP	Multipotent Progenitor
pDC	Plasmacytoid Dendritic Cell
UNK	Unknown (derived from bone marrow)

List of the cell lines used in the Buenrostro et al., 2018 data set, including the corresponding acronyms used to describe and label them.
