# Supplementary material for: Matrix factorization and transfer learning uncover regulatory biology across multiple single-cell ATAC-seq data sets
Source: Nucleic Acids Res. 2020 May 11;48(12):e68. doi: 10.1093/nar/gkaa349 (PMC7337516; doi:10.1093/nar/gkaa349)
Supplement: gkaa349_Supplemental_Files [file gkaa349_supplemental_files.zip › SupplementalFile2.pdf]

TF Enrichment

```
## $`Pattern 1`
##             (Other)             ZNF263          GATA1::TAL1
##                 961                801                  404
##                EGR1                SP2                 CTCF
##                 379                378                  296
##               RREB1          MAF::NFE2                  SP1
##                 211                184                  175
##                KLF5                SP4                FOXP1
##                 153                144                  135
##                IRF1              GATA2                FOSL1
##                 132                110                  109
##               STAT1               NFE2                 E2F6
##                  98                 94                   86
##                JUND              PLAG1                GATA3
##                  78                 77                   74
##               NR2C2 SMAD2::SMAD3::SMAD4                FOSL2
##                  72                 71                   68
##          NFIC::TLX1              KLF16                HNF4G
##                  65                 62                   61
##                PAX5        JUN(var.2)                 NFYA
##                  61                 60                   59
##              ZBTB18                YY1                ESRRB
##                  59                 57                   55
##                ZEB1              TBX15                STAT3
##                  55                 54                   53
##               PRDM1              INSM1                 REST
##                  52                 51                   51
##                ESR2             POU2F2          STAT1::STAT2
##                  48                 48                   48
##          RARA::RXRA                SP8                MEF2C
##                  46                 45                   44
##              SREBF1              NR2F1                 NRF1
##                  44                 43                   42
##       TFAP2B(var.2)               USF2                 SPIC
##                  41                 41                   37
##           BATF::JUN               CDX2          JUND(var.2)
##                  36                 35                   35
##                LEF1               NFYB                NHLH1
##                  35                 35                   35
##                RELA             SREBF2            TAL1::TCF3
##                  35                 34                   34
##                 JUN              KLF14                 ZIC3
##                  33                 33                   33
##                 MSC             ZNF740                 JDP2
##                  32                 32                   31
##                MAFK              FOXA1                  FOS
##                  31                 30                   28
##                EBF1         EWSR1-FLI1               POU1F1
##                  27                 26                   25
##                MAFF      TFAP2B(var.3)                CEBPA
##                  23                 23                   22
```

```
##              MEF2A              RUNX3       TFAP2A(var.3)
##                 22                 22                  22
##               USF1              TFAP4                DUX4
##                 22                 21                  20
##               ELK4           MAX::MYC         RORA(var.2)
##                 20                 20                  20
##             TCF7L2              TGIF2                E2F4
##                 20                 20                  19
##              FOXB1              FOXH1       TFAP2C(var.3)
##                 19                 19                  19
##               ZIC4              NFKB2                JUNB
##                 19                 18                  17
##        RARA(var.2)               SPI1                ZIC1
##                 17                 17                  17
##              FOXP2               ATF4              HOXB13
##                 16                 15                  15
##               HSF2
##                 15
##
## $`Pattern 2`
##            (Other)               IRF1              ZNF263
##               1219                592                 497
##              FOXP1         GATA1::TAL1              GATA2
##                487                460                 265
##              MEF2C               SPIC                CTCF
##                263                231                 228
##               SPI1              GATA3         STAT1::STAT2
##                189                182                 166
##              STAT1           MAF::NFE2          JUN(var.2)
##                163                157                 152
##             POU2F2              PRDM1                NFE2
##                152                146                 141
##               CDX2           BATF::JUN               FOXC2
##                123                113                 101
##              FOXA1              RREB1                JUND
##                 98                 97                  96
##               DUX4             POU1F1               RUNX3
##                 86                 86                  81
##              FOXB1              MEF2A               FOSL1
##                 80                 78                  77
##             ZBTB18         JUND(var.2)                JUN
##                 77                 73                  71
##               MAFK             POU4F3                 FOS
##                 71                 68                  66
##             TCF7L2            ONECUT3              POU4F1
##                 66                 64                  64
##             POU3F3              CEBPA               ESRRB
##                 63                 62                  62
##              HNF1A               JDP2                NFYA
##                 61                 61                  59
##              PROP1              STAT3               FOXP2
##                 59                 58                  57
##              FOXF2 SMAD2::SMAD3::SMAD4                LEF1
##                 55                 54                  53
```

```
##                HOXB13          RORA(var.2)                 MAFF
##                    49                   49                   45
##                  NFYB           EWSR1-FLI1                HNF4G
##                    45                   44                   44
##                 NFIL3                 EGR1                  YY1
##                    44                   43                   43
##                 FOXH1           TAL1::TCF3                FOSL2
##                    42                   41                   40
##                   MSC               POU3F4           RARA::RXRA
##                    40                   40                   40
##            NFIC::TLX1                NR2F1                FOXO3
##                    38                   38                   37
##                  PAX5                 USF2                 IRF2
##                    37                   37                   35
##                  PBX1                 HSF2                NR2C2
##                    35                   33                   33
##                POU3F2               POU4F2               POU6F2
##                    33                   33                   33
##                  MAFG              BHLHE23                 DUXA
##                    32                   31                   31
##                  IRF7               PHOX2A               HOXD13
##                    31                   30                   29
##                  HSF4                  TEF                 RELA
##                    27                   26                   25
##                 TGIF2                FOXC1                 REST
##                    25                   24                   24
##                 TBX15                 ETV2                MEF2B
##                    24                   23                   23
##                   SP2                  SP1                 MYF6
##                    23                   22                   21
##                  ATF4                 ESR2                RUNX2
##                    20                   20                   20
##                   SRF
##                    20
##
## $`Pattern 3`
##               (Other)                 IRF1               ZNF263
##                  1940                 1215                  735
##          STAT1::STAT2                FOXP1                MEF2C
##                   541                  501                  369
##                POU2F2                PRDM1                 CTCF
##                   311                  282                  252
##                 RREB1            BATF::JUN                 RELA
##                   216                  208                  193
##                 HNF1A           JUN(var.2)                 EGR1
##                   189                  182                  178
##                  NFE2            MAF::NFE2                 SPIC
##                   161                  154                  150
##                  SPI1                 CDX2                  SP2
##                   141                  118                  118
##                 MEF2A                 PAX5                TBX15
##                   116                  115                  107
##                  JDP2               TCF7L2               POU1F1
##                    95                   94                   91
```

```
##            HNF4G              LEF1             STAT1
##               89                89                89
##           ZBTB18              IRF2            POU4F1
##               89                86                86
##            FOXB1              DUX4              EBF1
##               85                82                82
##             IRF7             PROP1 SMAD2::SMAD3::SMAD4
##               81                81                79
##            GATA2             FOXC2             ESRRB
##               77                76                75
##       RORA(var.2)       JUND(var.2)               JUN
##               74                73                72
##             JUND             NFKB2            POU3F3
##               72                72                72
##            RUNX3             HNF1B               FOS
##               72                68                67
##            FOXA1       GATA1::TAL1             FOSL1
##               67                67                64
##             MAFK             STAT3              NFYA
##               64                63                61
##           POU4F3             CEBPA             FOXP2
##               60                59                58
##             ZEB1               MSC               YY1
##               58                56                56
##            NFIL3        TAL1::TCF3             GATA3
##               55                55                54
##           POU3F4               SP1           ONECUT3
##               53                53                48
##             NFYB              TBR1             FOXH1
##               47                47                45
##             MAFF             MEF2D            POU3F2
##               45                45                44
##             USF2             NR2C2              ESR2
##               44                43                42
##             KLF5             MEF2B             FOXO3
##               42                42                41
##            INSM1              IRF8             NR2F1
##               41                41                41
##             PBX1        RARA::RXRA            POU4F2
##               41                41                40
##              SP4             TBX20             TBX21
##               40                40                40
##       EWSR1-FLI1            HOXB13              NRF1
##               39                39                39
##             ELF3             FOSL2         NFIC::TLX1
##               38                38                37
##             MYF6             EOMES            POU6F2
##               36                35                35
##             E2F6
##               34
##
## $`Pattern 4`
##          (Other)            ZNF263             FOXP1
##             1210               541               386
```

```
##              IRF1          JUN(var.2)                NFE2
##               327                 325                 264
##         MAF::NFE2                JUND               MEF2C
##               244                 241                 232
##         BATF::JUN               FOSL1                 FOS
##               223                 222                 211
##             FOSL2              POU2F2               STAT1
##               156                 122                 122
##             RREB1                JDP2        STAT1::STAT2
##               118                 116                 114
##              CDX2               PRDM1                 JUN
##               113                 107                 100
##              EGR1               MEF2A               GATA2
##                98                  94                  90
##             FOXA1               FOXB1               FOXC2
##                88                  88                  88
##            ZBTB18               CEBPA         JUND(var.2)
##                88                  86                  86
##             STAT3                DUX4                SPIC
##                80                  67                  62
##             FOXP2         GATA1::TAL1               GATA3
##                59                  59                  57
##            POU4F3                EBF1                MAFK
##                57                  56                  55
##             PROP1                 SP2               FOXF2
##                53                  53                  52
##             HNF1A              TCF7L2               RUNX3
##                52                  51                  50
##         TAL1::TCF3                HSF1               NFIL3
##                50                  49                  48
##       RORA(var.2)               FOXH1               NR2C2
##                48                  47                  47
##            POU4F1 SMAD2::SMAD3::SMAD4       TFAP2B(var.2)
##                47                  47                  47
##              RELA               TBX15                CTCF
##                46                  46                  45
##              LEF1               ESRRB               FOXO3
##                45                  44                  43
##             HNF4G              HOXB13                ATF4
##                43                  43                  42
##            POU1F1                HSF2              POU3F3
##                40                  39                  39
##         RARA::RXRA                KLF5              POU4F2
##                39                  38                  38
##         EWSR1-FLI1                MAFF               MEF2B
##                37                  37                  37
##              NFYB                USF2                 MSC
##                37                  36                  35
##             MEF2D                E2F6                 SP1
##                34                  32                  31
##              PAX5              POU3F2       TFAP2B(var.3)
##                29                  29                  29
##         NFIC::TLX1                ESR2                JUNB
##                28                  27                  27
```

```
##              PLAG1              POU6F2                RFX3
##                 27                  27                  27
##              TFAP4              HOXC13               INSM1
##                 27                  26                  26
##              NR2F1             ONECUT3                MYF6
##                 26                  26                  25
##              TEAD3                 YY1              HOXD13
##                 25                  25                  24
##               HSF4                IRF2              POU3F4
##                 24                  24                  24
##                SRF
##                 24
##
## $`Pattern 5`
##               CTCF              ZNF263             (Other)
##               1748                1411                 930
##                SP2                EGR1                 SP1
##                922                 755                 294
##              RREB1                 SP4                KLF5
##                286                 233                 191
##               REST                E2F6                NRF1
##                165                 143                 135
##              PLAG1                ZEB1               NHLH1
##                122                  92                  85
##              KLF16               INSM1                NFYA
##                 83                  78                  76
##             POU2F2 SMAD2::SMAD3::SMAD4                PAX5
##                 73                  72                  67
##              NR2C2       TFAP2B(var.2)                IRF1
##                 66                  64                  61
##              STAT1               FOXP1                NFYB
##                 61                  59                  58
##      TFAP2B(var.3)                RFX3               KLF14
##                 58                  56                  54
##         EWSR1-FLI1                RELA                ESR2
##                 52                  51                  50
##              PRDM1                RFX5                EBF1
##                 50                  50                  49
##              STAT3               HNF4G                ZIC3
##                 49                  48                  47
##               E2F4         STAT1::STAT2       TFAP2A(var.3)
##                 45                  43                  43
##               ZIC4          NFIC::TLX1               TBX15
##                 43                  42                  40
##             ZBTB18            MAF::NFE2                 MSC
##                 40                  39                  39
##              NFKB2       TFAP2A(var.2)                USF2
##                 38                  37                  37
##                YY1              POU1F1       TFAP2C(var.3)
##                 37                  36                  36
##             ZNF740                RFX2                 SP8
##                 36                  34                  34
##             SREBF1               ESRRB               FOSL1
##                 32                  31                  30
```

```
##          RARA::RXRA                SPIC               NR2F1
##                  29                  29                  28
##          JUN(var.2)          TAL1::TCF3              TCF7L2
##                  27                  26                  26
##               GLIS2               HINFP                EGR3
##                  25                  25                  24
##               GATA2                 JUN         RORA(var.2)
##                  24                  24                  24
##               FOSL2                LEF1               FOXH1
##                  23                  23                  22
##         GATA1::TAL1              TFAP2B                ZIC1
##                  22                  22                  22
##           BATF::JUN              SREBF2                MAFK
##                  21                  21                  20
##              POU3F4                USF1              ZNF143
##                  20                  20                  20
##                CDX2               FOXP2                GLI2
##                  19                  19                  19
##               TFAP4                JUND                RFX4
##                  19                  18                  18
##                ELK4                 ID4         JUND(var.2)
##                  17                  17                  17
##               KLF13                EGR4                 FOS
##                  17                  16                  16
##            MAX::MYC              ZBTB7A             BHLHE41
##                  16                  16                  15
##                EGR2
##                  15
##
## $`Pattern 6`
##             (Other)              ZNF263                CTCF
##                4404                3142                3019
##                 SP2                EGR1                IRF1
##                1107                1096                 771
##                SPIC               RREB1               FOXP1
##                 648                 616                 480
##                 SP1                SPI1           MAF::NFE2
##                 472                 442                 363
##        STAT1::STAT2                KLF5                NRF1
##                 359                 343                 325
##               PRDM1                E2F6                 SP4
##                 305                 273                 259
##               MEF2C               STAT1              ZBTB18
##                 258                 245                 241
##                PAX5          JUN(var.2)               PLAG1
##                 238                 226                 225
##                NFE2               CEBPA                 YY1
##                 224                 222                 221
## SMAD2::SMAD3::SMAD4         GATA1::TAL1                ZEB1
##                 213                 211                 209
##              POU2F2               ESRRB               TBX15
##                 205                 192                 172
##               NR2C2               FOSL1               INSM1
##                 171                 169                 169
```

```
##              JUND              REST     TFAP2B(var.2)
##               165               163               163
##             STAT3              EBF1             NHLH1
##               156               154               154
##         BATF::JUN        NFIC::TLX1               JUN
##               151               151               143
##              ESR2             KLF16             NR2F1
##               142               141               141
##             HNF4G              RELA             RUNX3
##               140               137               134
##              USF2             GATA2              NFYA
##               133               132               131
##             FOSL2              ATF4             GATA3
##               130               129               125
##               MSC       JUND(var.2)     TFAP2B(var.3)
##               125               121               121
##              CDX2             FOXH1            TCF7L2
##               119               115               115
##              NFYB        EWSR1-FLI1             MEF2A
##               113               111               111
##        RARA::RXRA            ZNF740               FOS
##               109               105               103
##              MAFK              DUX4             FOXA1
##               101               100                97
##             FOXP2        TAL1::TCF3             FOXB1
##                97                96                94
##             KLF14              ELK4            SREBF1
##                93                92                89
##       RORA(var.2)            POU1F1              LEF1
##                88                86                82
##     TFAP2A(var.3)             FOXC2              ZIC3
##                80                79                79
##            SREBF2              MAFF             NFKB2
##                76                75                75
##               SP8              ELK1              RFX5
##                72                71                71
##          MAX::MYC              MYF6              JDP2
##                70                70                69
##            POU4F1           ONECUT3              IRF2
##                69                68                67
##              ZIC1              E2F4            HOXB13
##                67                66                66
##              ELF3
##                65
##
## $`Pattern 7`
##           (Other)            ZNF263              IRF1
##              1620              1063               621
##              CTCF              SPIC               SP2
##               503               371               353
##              EGR1      STAT1::STAT2             FOXP1
##               328               295               240
##              SPI1             RREB1         MAF::NFE2
##               231               208               161
```

```
##                PRDM1                MEF2C                CEBPA
##                  160                  150                  139
##                  SP1                  SP4                 NRF1
##                  128                  125                  115
##               POU2F2           JUN(var.2)                 KLF5
##                  113                   96                   96
##                  JUN                  YY1                FOSL1
##                   90                   88                   84
##                STAT1                 ATF4                NR2C2
##                   80                   76                   76
##                ESRRB                 CDX2               ZBTB18
##                   75                   74                   74
##                HNF4G                TBX15                 NFE2
##                   72                   72                   70
##                MEF2A                 NFYA             BATF::JUN
##                   68                   65                   63
##                 RELA                 PAX5 SMAD2::SMAD3::SMAD4
##                   63                   62                   62
##                FOSL2          JUND(var.2)                 NFYB
##                   61                   61                   60
##                 E2F6                 JUND                 USF2
##                   58                   56                   56
##                PLAG1                INSM1           EWSR1-FLI1
##                   55                   53                   52
##                FOXB1                NR2F1                STAT3
##                   52                   52                   52
##                 ZEB1                FOXA1                RUNX3
##                   50                   49                   48
##                 MAFF                FOXH1                 MAFK
##                   47                   46                   46
##                NFIL3           RARA::RXRA            NFIC::TLX1
##                   46                   46                   45
##                 REST               TCF7L2                 DUX4
##                   45                   45                   44
##                  FOS                FOXP2                GATA2
##                   43                   41                   41
##          RORA(var.2)                  TEF                 ESR2
##                   41                   41                   40
##                FOXC2        TFAP2B(var.2)        TFAP2B(var.3)
##                   40                   40                   40
##                NFKB2                NHLH1                 RFX3
##                   39                   39                   38
##                  MSC               POU1F1                 LEF1
##                   37                   36                   35
##                 HSF4                 IRF2                PROP1
##                   33                   33                   32
##                 RFX5           TAL1::TCF3                FOXF2
##                   32                   32                   31
##          GATA1::TAL1                 HSF1                KLF16
##                   31                   31                   31
##                MEF2B               SREBF2                 PBX1
##                   31                   31                   30
##               POU4F1                 EBF1                 IRF7
##                   30                   29                   29
```

```
##                 KLF14             POU4F3              TFAP4
##                    29                 29                 29
##                  ELF3             POU3F3              GATA3
##                    28                 28                 27
##                  JDP2
##                    27
```
