# Supplementary material for: Matrix factorization and transfer learning uncover regulatory biology across multiple single-cell ATAC-seq data sets
Source: Nucleic Acids Res. 2020 May 11;48(12):e68. doi: 10.1093/nar/gkaa349 (PMC7337516; doi:10.1093/nar/gkaa349)
Supplement: gkaa349_Supplemental_Files [file gkaa349_supplemental_files.zip › SupplementalFile3.pdf]

# CoGAPS + GREAT Hallmark Pathways

```
## $`Pattern 1`
## $`Pattern 1`$summaryTable
##                       pathway       PValue
## 2     HALLMARK_HEME_METABOLISM 2.600580e-06
## 1     HALLMARK_APICAL_JUNCTION 5.432028e-05
## 3 HALLMARK_IL2_STAT5_SIGNALING 5.432028e-05
##
##
## $`Pattern 2`
## $`Pattern 2`$summaryTable
## NULL
##
##
## $`Pattern 3`
## $`Pattern 3`$summaryTable
##                             pathway       PValue
## 7   HALLMARK_TNFA_SIGNALING_VIA_NFKB 7.585408e-16
## 3       HALLMARK_IL2_STAT5_SIGNALING 2.790409e-12
## 1       HALLMARK_ALLOGRAFT_REJECTION 1.274851e-10
## 6 HALLMARK_INTERFERON_GAMMA_RESPONSE 2.661608e-08
## 5     HALLMARK_INFLAMMATORY_RESPONSE 1.410899e-07
## 4   HALLMARK_IL6_JAK_STAT3_SIGNALING 1.411458e-06
## 2   HALLMARK_ESTROGEN_RESPONSE_EARLY 5.924154e-05
##
##
## $`Pattern 4`
## $`Pattern 4`$summaryTable
##                                     pathway       PValue
## 2 HALLMARK_EPITHELIAL_MESENCHYMAL_TRANSITION 2.521453e-14
## 5                   HALLMARK_UV_RESPONSE_DN 1.448222e-09
## 4                         HALLMARK_HYPOXIA 5.066549e-07
## 1                  HALLMARK_APICAL_JUNCTION 3.980659e-06
## 3          HALLMARK_ESTROGEN_RESPONSE_EARLY 2.819383e-05
##
##
## $`Pattern 5`
## $`Pattern 5`$summaryTable
## NULL
##
##
## $`Pattern 6`
## $`Pattern 6`$summaryTable
##                             pathway       PValue
## 13 HALLMARK_TNFA_SIGNALING_VIA_NFKB 1.695998e-12
## 11             HALLMARK_P53_PATHWAY 2.733444e-09
```

```
## 6   HALLMARK_ESTROGEN_RESPONSE_LATE 5.113971e-07
## 7                  HALLMARK_HYPOXIA 5.113971e-07
## 8      HALLMARK_IL2_STAT5_SIGNALING 1.708354e-06
## 10   HALLMARK_INFLAMMATORY_RESPONSE 1.708354e-06
## 9  HALLMARK_IL6_JAK_STAT3_SIGNALING 1.888954e-06
## 5  HALLMARK_ESTROGEN_RESPONSE_EARLY 5.467206e-06
## 12 HALLMARK_PI3K_AKT_MTOR_SIGNALING 8.814143e-05
## 1      HALLMARK_ALLOGRAFT_REJECTION 1.367897e-04
## 2          HALLMARK_APICAL_JUNCTION 1.367897e-04
## 4               HALLMARK_COMPLEMENT 1.367897e-04
## 3                HALLMARK_APOPTOSIS 1.532638e-04
##
##
## $`Pattern 7`
## $`Pattern 7`$summaryTable
##                               pathway       PValue
## 12   HALLMARK_TNFA_SIGNALING_VIA_NFKB 6.898088e-30
## 8      HALLMARK_INFLAMMATORY_RESPONSE 3.939475e-21
## 7    HALLMARK_IL6_JAK_STAT3_SIGNALING 9.414542e-13
## 1        HALLMARK_ALLOGRAFT_REJECTION 7.104207e-11
## 2                  HALLMARK_APOPTOSIS 2.972834e-09
## 6        HALLMARK_IL2_STAT5_SIGNALING 1.634058e-08
## 9  HALLMARK_INTERFERON_GAMMA_RESPONSE 8.944123e-08
## 11               HALLMARK_P53_PATHWAY 4.608754e-07
## 13            HALLMARK_UV_RESPONSE_UP 2.909832e-06
## 5                    HALLMARK_HYPOXIA 1.009411e-05
## 3                 HALLMARK_COMPLEMENT 4.262823e-05
## 4    HALLMARK_ESTROGEN_RESPONSE_EARLY 1.672786e-04
## 10         HALLMARK_KRAS_SIGNALING_UP 1.672786e-04
```
